# Simulating the Transmural Mechanical Response of Functionally Graded Arterial Grafts

**DOI:** 10.1021/acsabm.5c01506

**Published:** 2025-11-15

**Authors:** Katie L. Fegan, Amy V. Tansell, Asif J. Iqbal, Lauren E.J. Thomas-Seale

**Affiliations:** † Physical Sciences for Health Centre for Doctoral Training, 1724 University of Birmingham, Birmingham B15 2TT, U.K.; ‡ Department of Mechanical Engineering, University of Birmingham, Birmingham B15 2TT, U.K.; § School of Mathematics, University of Birmingham, Birmingham B15 2TT, U.K.; ∥ Department of Cardiovascular Sciences, University of Birmingham, Birmingham B15 2TT, U.K.

**Keywords:** cardiovascular disease, synthetic graft design, Poly(vinyl alcohol)/Gelatin cryogels, vessel mimicking materials, transmural mechanical response, finite element analysis

## Abstract

With coronary artery disease remaining the leading cause of mortality worldwide, the design and manufacture of clinically viable synthetic coronary artery grafts remains a fundamental healthcare challenge. It is widely accepted that vascular mimicking materials (VMMs) should emulate the heterogeneous biomechanical and biological functions of the multilayered artery wall to ensure long-term patency postimplantation. However, few VMMs can adequately meet these complex design requirements. Poly­(vinyl alcohol) (PVA)/gelatin cryogels are prospective VMMs due to their combined mechanical (PVA) and biointegrative (gelatin) features, but their development thus far has been limited to homogeneous constructs. The aim of this research is to assess the mechanical response of biomimetically designed multilayered grafts, simulated using Finite Element Analysis. The impact of a sinusoidal interface on circumferential stress distribution and graft compliance, was explored. Using qualitative insight from research on hydrogel based functionally graded biomaterials, and in the context of subzero extrusion additive manufacturing, rough (infinite) friction was used to model the contact between the layer. It was found that transmural stress patterns were continuously graded (phased) as a function of interface amplitude and frequency. In contrast to laminated models, which displayed a discontinuity in transmural stress between layers. This design methodology illustrates a novel approach to achieving functionally graded synthetic grafts through interface design.

## Graphical abstract

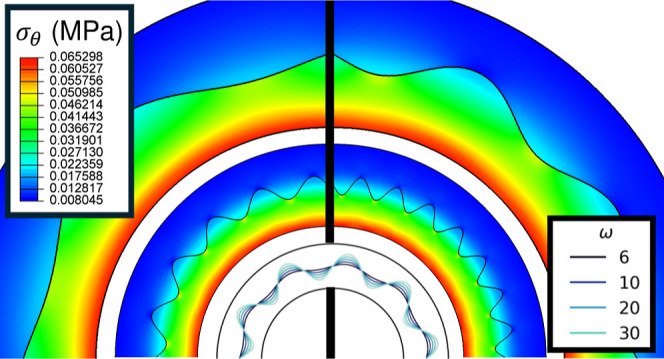

## 1 Introduction

Cardiovascular disease (CVD) is the leading cause of death worldwide. Despite increasing awareness of the behavioral and metabolic risk factors associated with CVDs, they are responsible for almost a third of global deaths reported annually.^1^ Due to rising healthcare costs and increasing years lived with disability, treating CVD is listed as a major sustainable development goal by the United Nations and the World Health Organization.^2,3^ The common denominator shared by many CVDs is the narrowing and occlusion of vessels that supply blood to various regions of the body. Of these, coronary artery disease, or CAD, is the most prevalent: in 2019, CAD alone killed more than nine million people worldwide.^1^ Patients with advanced CAD often require bypass surgery to re-establish blood flow to the heart and prevent further downstream complications, including heart failure, heart attack and death.

Synthetic grafts must support the native biological functions of the coronary artery wall.^4^ Mechanical mismatch between a graft and its host vessel has long been cited as a major cause of graft failure due to the complex interplay between vascular cells and their surrounding mechanical environment.^5^ Poly­(vinyl alcohol) (PVA) cryogel demonstrates cytocompatibility; it is a hydrophilic vascular mimicking material (VMM) with tunable mechanical properties and is thus a compelling biomaterial across a spectrum of tissue engineering applications.^6,7^ Significant effort has been made to manufacture PVA composites with improved biological integration; in cardiovascular tissue engineering, PVA/gelatin cryogel has demonstrated enhanced endothelialisation and hemocompatibility with respect to pure PVA.^8,9^

To date, PVA/gelatin has only been applied to the manufacture of homogeneous constructs. Yet, arteries, among other biological tissues, achieve multifunctional combinations of strength, stiffness and toughness through a combination of material and geometric heterogeneity.^10^ It follows that research into PVA/gelatin cryogel as a VMM should progress into the realm of heterogeneous graft design, drawing on biomimetic concepts such as functional grading.^11^ With the advent of advanced manufacturing techniques, such as subzero additive manufacturing (AM),^12–15^ more complex graft design may offer mechanical characteristics that mimic the nature arterial wall more closely.

While computational modeling has become a staple tool to simulate hemodynamic behavior in arteries, its application to the design and manufacture of soft synthetic vascular grafts is comparatively limited. Specifically, Finite Element Analysis (FEA)—a solid modeling technique used extensively in the design of hard tissue implants to evaluate the governing biomechanical interactions under physiological loading—is not routinely used in the assessment of novel VMM designs. Given the need to emulate the physiological functions of coronary arteries to prevent premature graft failure, FEA offers a complementary method to AM for exploring these design challenges in more depth, toward optimizing the stress distribution and compliance of different graft designs.^16^

This paper aims to assess the mechanical response of biomimetic graft design, based on the more complex geometric capabilities of AM. Drawing bioinspiration from the macroscale waviness of the elastic lamellae (Figure 1) and the microscale crimped waveform of collagen fibers, the impact of a sinusoidal interface, the radius, amplitude and frequency, on the transmural biomechanics of PVA/gelatin grafts will be assessed parametrically using FEA. In addition, the suitability of various approaches to model the contact behavior between complex interfaces will be explored. It is intended that the findings from this research will complement the future design and development of PVA-based grafts fabricated using AM. Furthermore, while PVA/gelatin has been chosen as the VMM material, it is anticipated that the approach to interface design presented in this study will translate more widely to the design of biomimetic coronary artery grafts.

**Figure 1:**
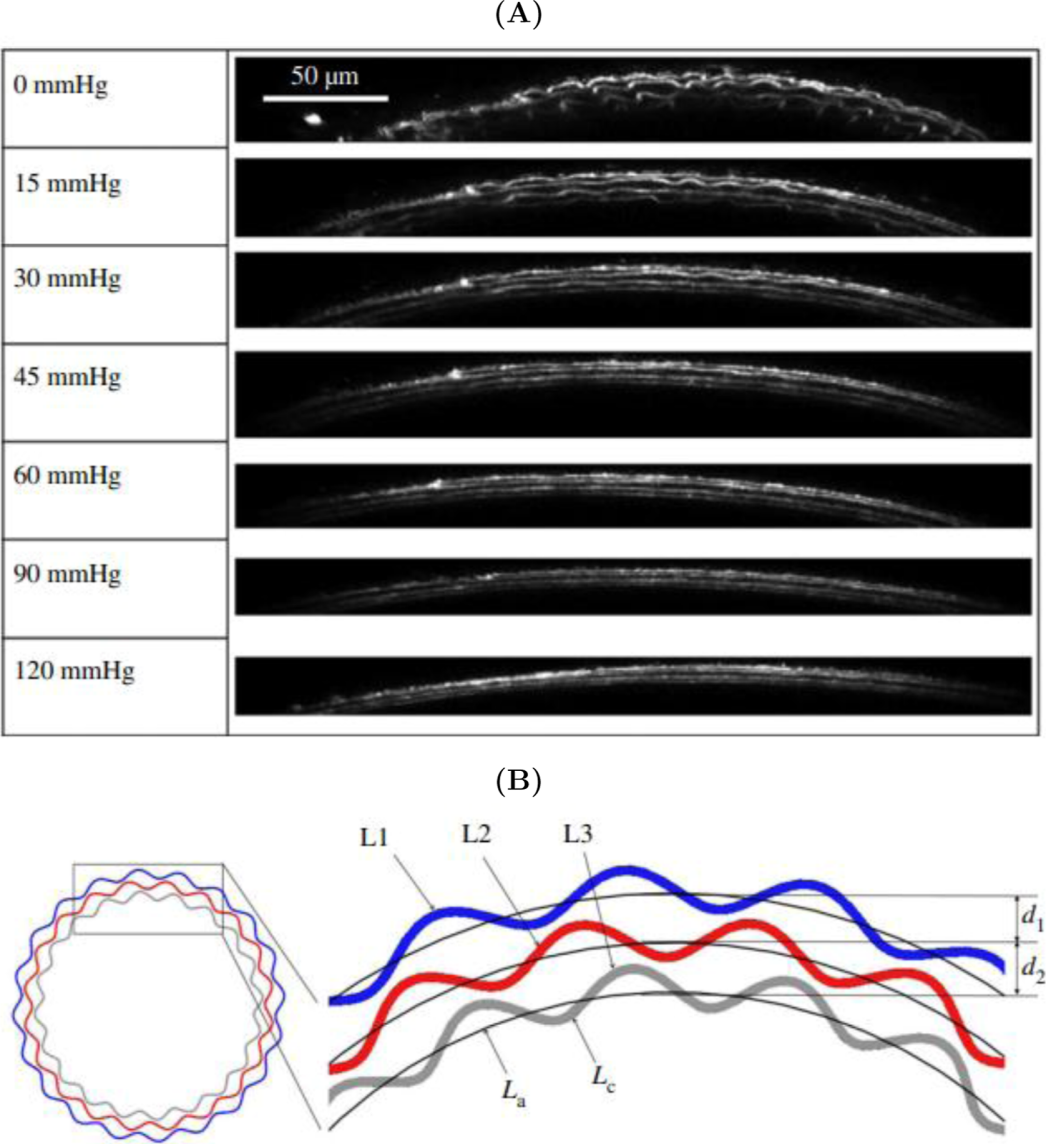
Micromechanics of elastic lamellae. (A) Transverse cross-sectional images of mouse carotid artery elastic lamellae when loaded up to systolic pressure and (B) Schematic of three elastic lamellae layers, L1, L2 and L3, in the arterial wall, with *L*_a_, the arc length, *L*_c_, the contour length, and *d*, the interlamellar distance. Both figures reprinted from ref 17 with permission from The Royal Society (UK).

## 2 Theory

Nature uses structural and hierarchical design elements to meet the strength, toughness and flexibility requirements of a given organ or tissue.^10,18^ The interfaces between regions of opposing properties play a vital role in determining the overall mechanical performance of a tissue in its biomechanical environment. For instance, the compliant interface between dentin and enamel in teeth improves the fracture toughness of the otherwise brittle enamel; in the skull, interdigitated suture lines provide both flexibility for growth and strength.^19,20^

In arteries, the interfaces between the three tissue layers are separated by elastic laminae. Not only are the internal (intima-media) and external (media-adventitia) elastic laminae approximately triple the stiffness of the media in healthy coronary arteries,^21^ but these undulating sheets straighten as they bear load under intraluminal pressure, helping to evenly distribute transmural stresses (Figure 1A).^17,22^ Moreover, the ‘waviness’ of the elastic lamellae is amplified toward the inner surface of the wall to compensate for increased circumferential stretch in the intima (Figure 1B).^17^ Thus, many biological tissues use variations in structure and composition to introduce gradated properties along at least one direction throughout their volume. This concept, known as functional grading, can be applied to biomaterials to create tissue implants with versatile mechanical and physical properties.^23^

The majority of functionally graded biomaterials (FGBMs) are designed for orthopedic^24,25^ and orthodontic^26^ applications. The capabilities of AM to vary internal structures and material composition or properties within a single part mean that it is a key enabler of FGBMs. While many FGBMs are comprised of thermoplastics, ceramics or metals,^24^ comparatively fewer soft FGBMs are reported in the literature, despite the need to replicate the multilayered structures of soft tissues such as arteries.^27^ The mismatch in mechanical properties between arterial grafts which are stiffer than their respective artery (such as Dacron grafts) fail due to intimal hyperplasia induced by compliance mismatch and subsequent turbulent flow.^5^ It is hypothesized that interfacing using FGBMs could alter the stress pattern at the prosthesis–tissue interface, reducing or redistributing stress concentrations when placed under external load.^24,28^

Layered grafts, such as those presented by Fegan et al., are examples of discontinuous FGBMs.^29^ The discrete interfaces between each layer create stepwise changes in composition and thus mechanical properties.^23^ By contrast, continuous FGBMs use gradual transitions between dissimilar materials to reduce moduli mismatch between interfacing materials.^28,30^ The parameters of freeze–thaw (FT) cycling have been explored to create PVA cryogel constructs with continuous functional grading. Wahab et al. created synthetic glenoid labrum implants by casting high- and low-stiffness PVA into a two-chamber mold.^31^ By removing the divider between both chambers and allowing diffusion between both solutions, they obtained a continuous FGBM with a stiffness gradient of 0.1–0.4 MPa that was capable of reducing the stress at the glenohumeral joint by approximately 50%.^31^ Alternatively, gradual directional freezing methods have been used to manufacture PVA cylinders with longitudinal stiffness gradients between 1 and 200 kPa.^32,33^

Ultimately, these methods lack control over the resulting gradient. The design flexibility afforded by technologies such as AM gives engineers the ability to manufacture FGBMs with biomimetic, interlocking interfaces at the mesoscale.^34–38^ While discontinuous in nature, the suture geometry can be designed to yield a homogeneous stress field and impart optimized load transmission, energy absorption and fatigue behavior.^39^ Subzero AM has been used to manufacture PVA-based cryogels with biomimetic gradients through control of the infill density^40^ and print toolpath.^41^ As such, subzero AM offers the capacity to fabricate heterogeneous PVA-based cryogels with more complex interfaces, to optimize the transmural stress distribution.

## 3 Method

### 3.1 Overview

The workflow of this study is provided in Figure 2. In the first instance, the boundaries between interfacing layers of interdigitated and laminated grafts were assessed according to three contact formulations. As outlined in Supporting Information (Section S2), this preliminary work was used to inform the interfacing boundary conditions in the main parametric study and to evaluate the impact of the sinusoidal interface parameters. All models were constructed in Abaqus/CAE 2021 (Dassault Systèmes Simulia Corp., Rhode Island, US) using a 2D quasi-static implicit analysis.

**Figure 2:**
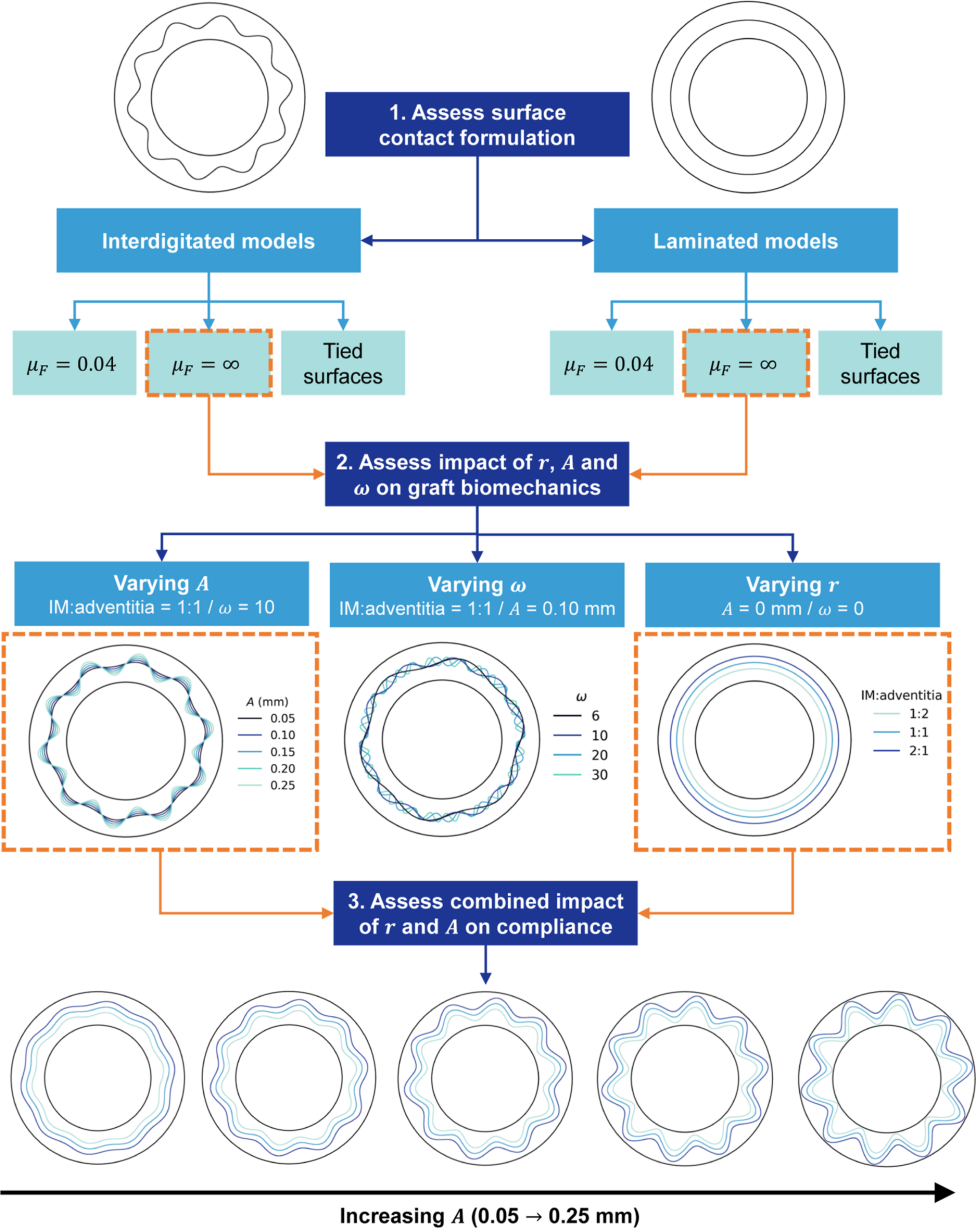
Workflow and variables under analysis, where μ_F_ denotes coefficient of friction and *r*, *A* and ω, denote the graft radius, amplitude and frequency (per 360° graft), respectively.

### 3.2 Finite Element Model

Bilayered, interdigitated PVA/gelatin grafts were generated using parametric equations to create a sinusoidal wave following a circular path of radius *r*

1 $$\begin{array}{l} x=\left(r+A\;\sin\left(\omega \theta \right)\cos\theta \right)+c_{x},\\ y=\left(r+A\;\sin\left(\omega \theta \right)\sin\theta \right)+c_{y}, \end{array}$$

where *c*_*x*_ and *c*_*y*_ are the central *x* and *y* coordinates of the circle, *A* and ω are the amplitude and frequency of the sine wave, respectively, and θ > 0 is the arc angle measured clockwise from the *y*-axis. As captured in (1), the interface geometry was therefore characterized by three independent variables: *A*, ω (per 360° graft) and *r*.

A bilayered model was chosen to reduce the complexity of the graft and allow investigation of the biomechanics of a single interdigitated interface. The innermost layer was representative of a combined intima-media (IM) layer and the outermost layer was representative of the adventitia in line with bilayered coronary artery models from the literature.^42,43^

The total wall thickness of the grafts were set to 0.87 mm to mirror the total thickness of the coronary artery wall.^44^ The impact of *r* on the compliance and the distribution of circumferential stress, σ_θ_, of the graft was analyzed by setting *A* and ω to zero and varying the relative thickness of the IM:adventitia (1:2, 1:1 and 2:1). The impact of *A* on the compliance and σ_θ_ distribution of the graft was individually assessed by keeping ω and *r* fixed; similarly, the impact of ω was tested by keeping *A* and *r* fixed. In both cases, *r* was set such that the relative thickness of the IM:adventitia was 1:1 (*r* = 1.815 mm). The graft geometry was surrounded by soft tissue to represent an artery embedded in connective tissue.^29^ The outer boundary of the soft tissue was constrained in all degress of freedom.

To establish the most appropriate method for modeling contact between complex interfaces, in this preliminary research, the following contact formulations were compared: μ_*F*_ of 0.04;^45^ rough friction, where μ_*F*_ = ∞; and tied surfaces. The impact of the three contact formulations were compared using a noninterdigitated model (*A* = 0 mm, ω = 0 and *r* = 1.815 mm) and an interdigitated model with a sinusoidal interface of *A* = 0.15 mm, ω = 10 and *r* = 1.815 mm. To distinguish between stress patterns relating to the interface and stress patterns relating to the choice of cryogel composition, each contact formulation was tested on a model where the material properties of the IM and adventitia were the same and a model where the IM was stiffer than the adventitia.

All grafts were partitioned into quarters and discretized using quadrilateral plane stress (CPS4R) elements. The structured mesh enabled transmural nodal analysis paths to be taken as a function of the phase, ϕ, of the sine wave. For example, consider a graft whose interface has a sinusoidal frequency of 10—if the circumference of the graft is seeded with 360 elements, then each period of the sine wave will be comprised of 36 elements, and the circumferential edge length of each element will correspond to a phase shift of 10°. This is displayed visually in Figure 3. In cases where the stress analyses of two or more models were compared and their interfaces were out of phase (such as when varying interface ω, as illustrated in Figure 4), the analyses were also conducted with respect to arc length or arc angle.

**Figure 3:**
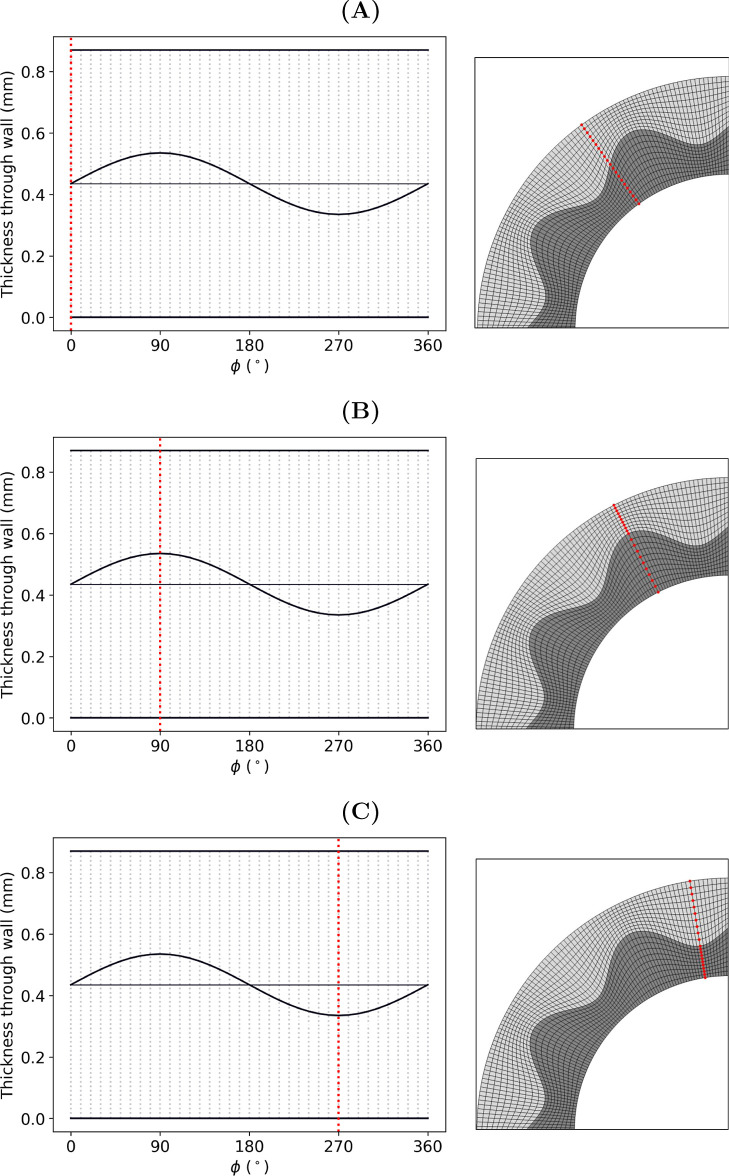
Examples of nodal analysis paths (red dotted lines) taken at phase, ϕ = (A) 0°, (B) 90° and (C) 270°. In the left panel, gray dotted vertical lines represent a circumferential mesh density of 36 elements/wave.

**Figure 4:**
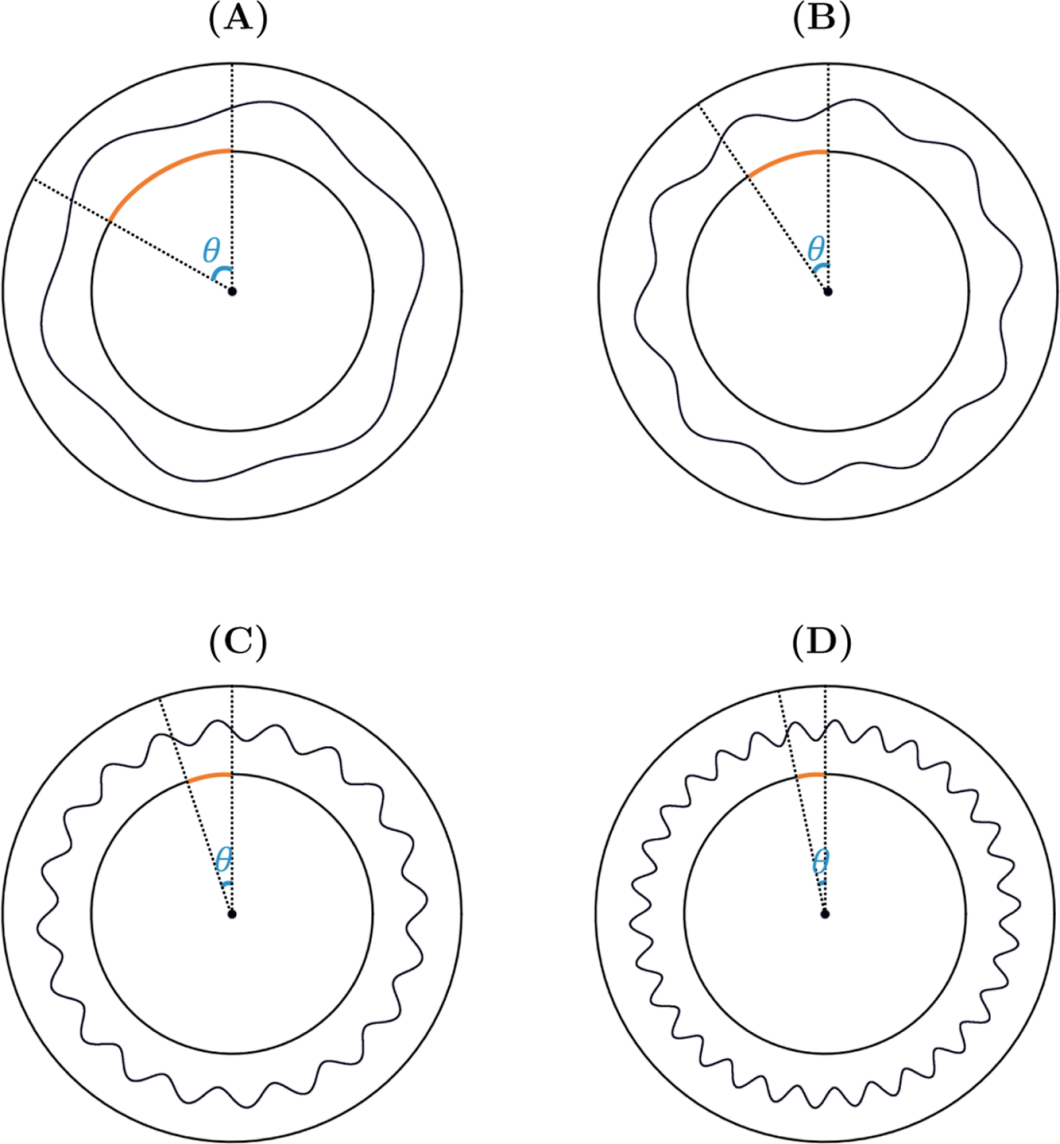
Interfaces comprised of different frequencies were out of phase with respect to each other. (A) ω = 6, (B) ω = 10, (C) ω = 20 and (D) ω = 30. Arc lengths are shown in orange and arc angles, θ, are shown in blue.

In addition to transmural stress analysis through the radial thickness of the wall, the variation in σ_θ_ along the interface was calculated using the nodal path highlighted in Figure 5. To account for in phase and out of phase interfaces (as determined by variation of *A* and ω), σ_θ_ was plotted as a function of ϕ and/or graft arc angle.

**Figure 5:**
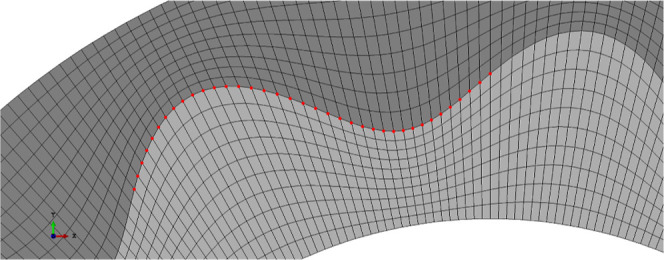
Node path corresponding to stress analyses along the interface. The stress at the nodes in red were plotted either as a function of phase, ϕ, or as a function of graft arc angle.

The mechanical properties of the two layers were modeled using the first order Ogden constitutive parameters and densities of the stiffest and most compliant PVA/gelatin compositions taken from ref 29 w/w ratio of PVA:gelatin of 9:1 and *M*_w_ 146–186 kDa with coagulation treatment (PG-A) and *M*_w_ 89–98 kDa without coagulation treatment (PG-B). These are listed in Table 1. The IM layer was set to the stiffer composition of PVA/gelatin (PG-A) and the adventitia was set to the more compliant composition (PG-B). The surrounding soft tissue was modeled as a Hookean linearly elastic material (Table 1), using an average value of density for soft tissue taken from the literature.^46^

**Table 1:** Material Parameters, Where *M*_w_ Denotes Weight Average Molecular Weight, μ_1_ and α_1_ Denote Ogden Parameters of 1st Order, *E* Denotes Young’s Modulus, ν Denotes Poisson’s Ratio, and ρ Denotes Density

material properties			
	PG-A^29^	PG-B^29^	**surrounding tissue** ^46^
***M***_**w**_ (**kDa**)	146–186	89–98	-
**coagulation** ^29^	yes	no	-
**μ**_**1**_ (**MPa**)	0.0444 ± 0.0060	0.0227 ± 0.0025	-
**α**_**1**_	7.116 ± 0.528	7.311 ± 0.581	-
*E*(**MPa**)	-	-	0.05
**ν**	-	-	0.49
**ρ** (**kg m** ^–**3**^)	1080 ± 36	1120 ± 38	1040

### 3.3 Compliance and Lumen Radii Analysis

As all graft models in this study were modeled in two-dimensions (2D), graft compliance was calculated as the cross-sectional compliance (CC = Δ*A*/Δ*P*, with units mm^2^/mmHg, where Δ*A* and Δ*P* denote change in area and pressure, respectively). CC may be used in clinical practice if axial vessel movement due to pulse pressure is assumed to be negligible compared with the change in vessel diameter.^47,48^ Understanding the impact of interdigitation on the CC of the graft was therefore determined to be a key measure of graft performance.

The presence of the interdigitated wave yielded nonuniform radial deformation around the lumen. Thus, the lumen diameter—and therefore Δ*A*—was dependent on where it was measured around the luminal surface. More specifically, the diameter varied as a function of the ϕ of the wave. Even-numbered frequencies (ω = 6, 10, 20, or 30 per 360° graft) were chosen to ensure that the phases of the wave on opposite sides of the graft were equivalent. This symmetry ensured that, for any given diameter, the graft expanded equally on both sides of the central coordinates of the model. For any given model, the CC was calculated as a function of ϕ; these CC values were then summed to give the mean CC (denoted $\overset{®}{CC}$ ) of the graft.

For the purpose of comparing the impact of interface parameters on graft compliance, $\overset{®}{CC}$ is presented as the absolute compliance between diastole and systole (mm^2^/mmHg). However, to allow additional comparison of PVA/gelatin graft compliance with the compliance of coronary arteries (as reported in the literature), the compliance was also normalized to the graft diameter at diastolic pressure (CC = (*D*_sys_ – *D*_dia_)/*D*_dia_ × 1/Δ*P* × 10^4^, with units %/100 mmHg, where *D*_sys_ and *D*_dia_ denote graft diameter at systolic and diastolic pressure, respectively).^49^

In addition to nonuniform $\overset{®}{CC}$ , the presence of nonuniform radial deformation around the lumen with pressure introduced a luminal surface wave. Plotting lumen radius, *r*_lumen_, as a function of ϕ, graft arc angle or graft arc length at a specified pressure allowed indirect mapping of the lumen surface profile. Due to the sinusoidal interface geometry, the surface height profile of the lumen was calculated by subtracting the minimum *r*_lumen_ value from the maximum *r*_lumen_ value.

It was hypothesized that a variation of sine wave parameters would impact the magnitude of $\overset{®}{CC}$ by changing the spatial ratio of the IM and adventitia through the graft and hence graft distensibility. Therefore, to investigate the impact of sine wave parameters on the spatial ratio, sectors of each graft were reconstructed in Fusion 360 (Autodesk, California, US) to approximate the area, as demonstrated in the Supporting Information (Figure S1).

The changes in $\overset{®}{CC}$ as a function of *A* and ω were analyzed in SigmaPlot 14.5 (Grafiti LLC, California, US) using one-way ANOVA. Note that, as only one diameter (and therefore *CC*) was obtained for each of the laminated models, statistical significance could not be obtained as a function of *r*.

## 4 Results

### 4.1 Overview

The results from this study are presented according to the order depicted in the study overview displayed in Figure 2. Due to the number of models developed in this study, modeling the contact between interdigitated interfaces is shown and discussed in the Supporting Information (Section S2). While the σ_θ_ and *x*_θ_ outputs from the rough friction and tied surface models were comparable, the rough friction model allowed analysis of the contact pressure at the interface (Figure S7). The laminated model, as shown in Figure 2, is the control study in this research. The impact of the interface radius on the stress distribution and compliance is presented in Supporting Information (Sections S3 and S4).

### 4.2 Impact of Interface Amplitude on Graft Biomechanics

#### 4.2.1 Varying Interface Amplitude: Stress Distribution

For all phases, the magnitude of σ_θ_ at the luminal surface increased with increasing *A* (Figures 6 and 7). Of the three phases presented in Figure 7, the difference in luminal σ_θ_ between the smallest (*A* = 0.05 mm) and largest (*A* = 0.25 mm) amplitudes ranged from 0.0017 MPa (ϕ = 270°) to 0.0048 MPa (ϕ = 0°).

**Figure 6:**
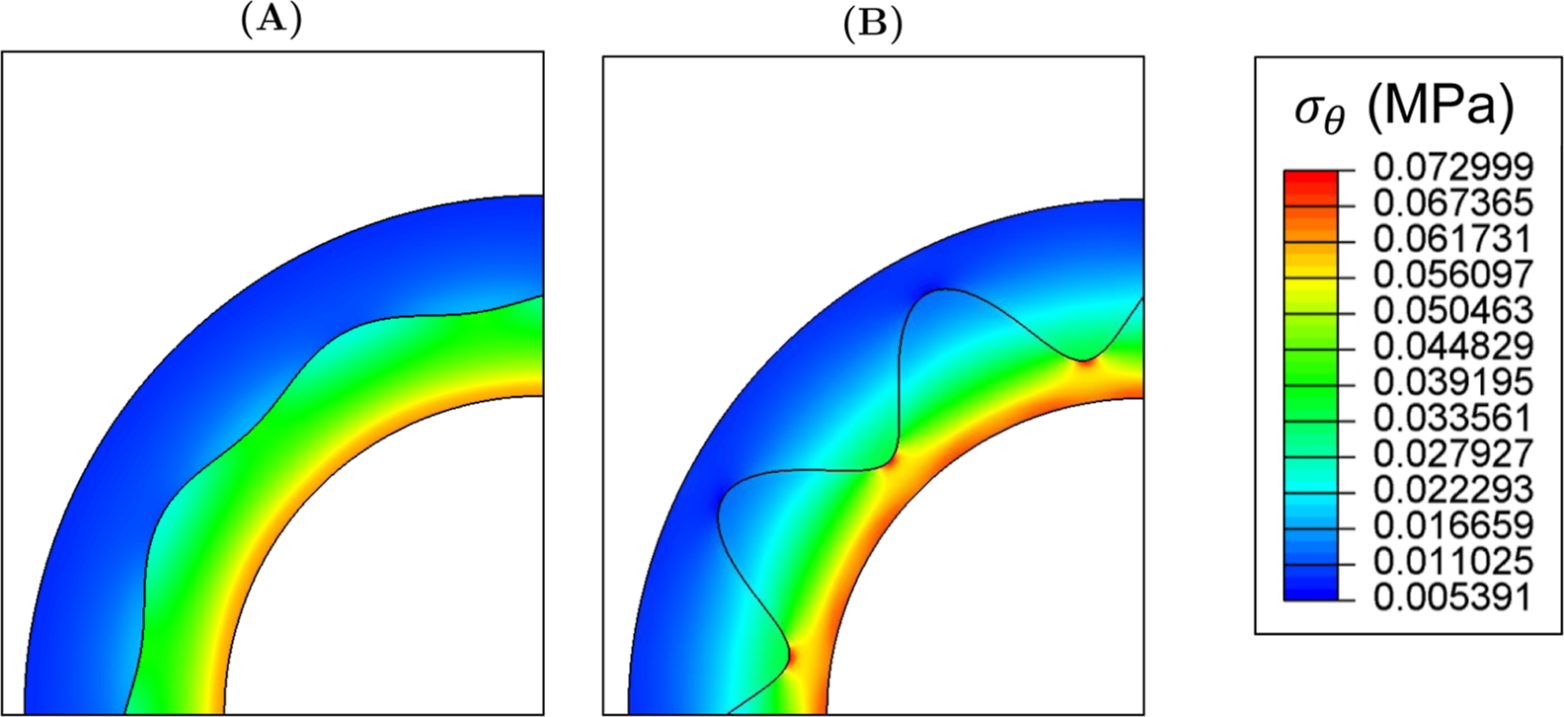
Distribution of circumferential stress, σ_θ_, at systolic pressure, at amplitude (A) A = 0.05 mm and (B) A = 0.25 mm.

**Figure 7:**
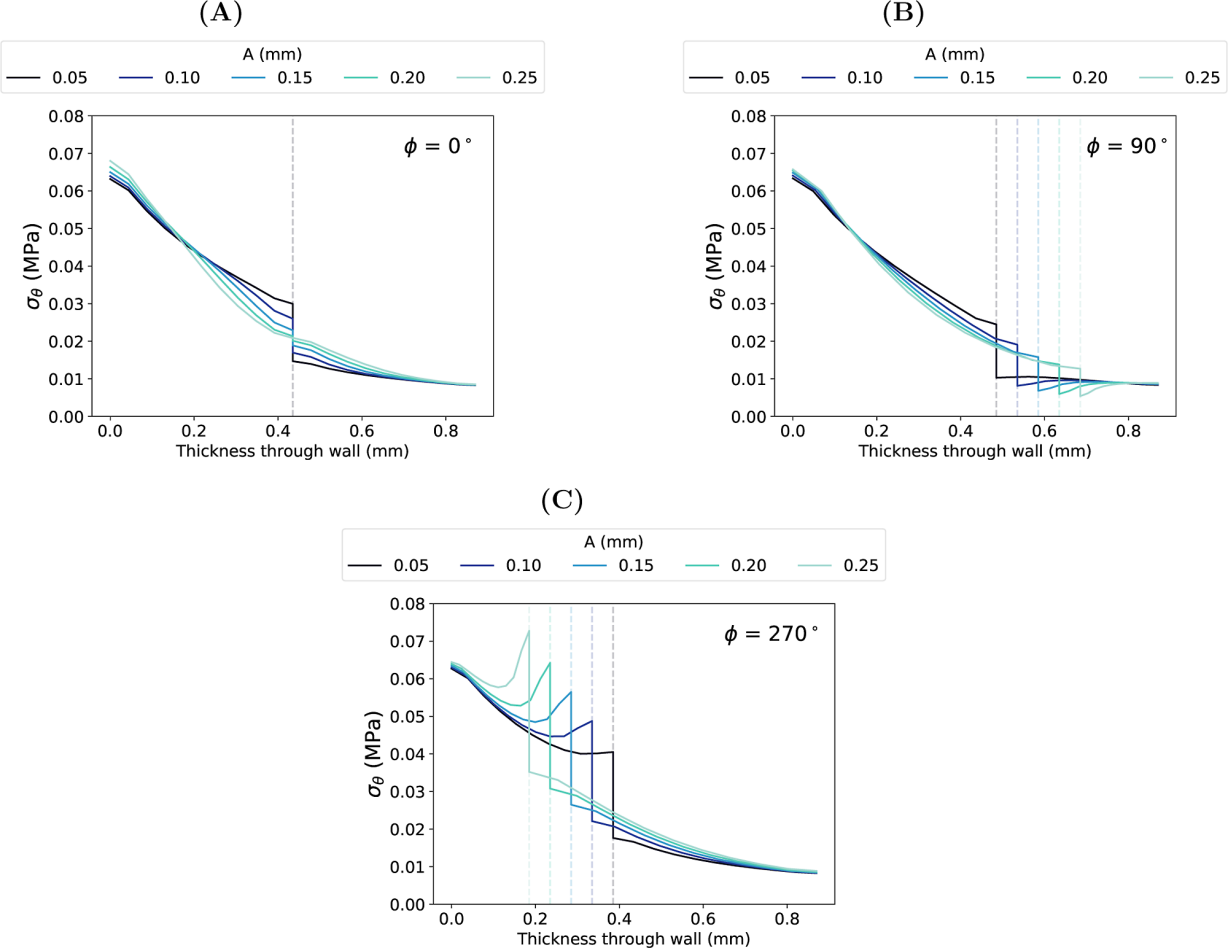
Distribution of transmural circumferential stress, σ_θ_, at systolic pressure with varying amplitude, *A*, at phase, ϕ equal to (A) 0°, (B) 90° and (C) 270°. Dashed lines show the interface at each ϕ.

When *A* ≤ 0.15 mm, the maximum σ_θ_ of each model was highest at the luminal surface across all phases and was followed by a marked decrease at the interface (Figure 7). The extent of this decrease correlated with increasing *A* (Figure 7). At ϕ = 0° and ϕ = 90°, the magnitude of σ_θ_ reduction at the interface decreased with increasing *A*, indicating a smoother transition in transmural stress across the interface at these phases when *A* was increased. For example, at systolic pressure, when *A* = 0.05 mm, the σ_θ_ at the interface decreased by 0.015 MPa at ϕ = 0° and 0.014 MPa at ϕ = 90°. When *A* = 0.25 mm, σ_θ_ decreased by 0.00006 and 0.007 MPa, respectively. However, at ϕ = 270°, the magnitude of σ_θ_ reduction at the interface increased with increasing amplitude. The distance between the lumen and the troughs of the wave decreased with increasing *A*; the IM was placed under higher tension in this region, as demonstrated by the increased stress concentration measured at this phase.

For all models, the highest and lowest interface contact pressures were observed at ϕ = 270° and 0°/180°, respectively (Figure 8).

**Figure 8:**
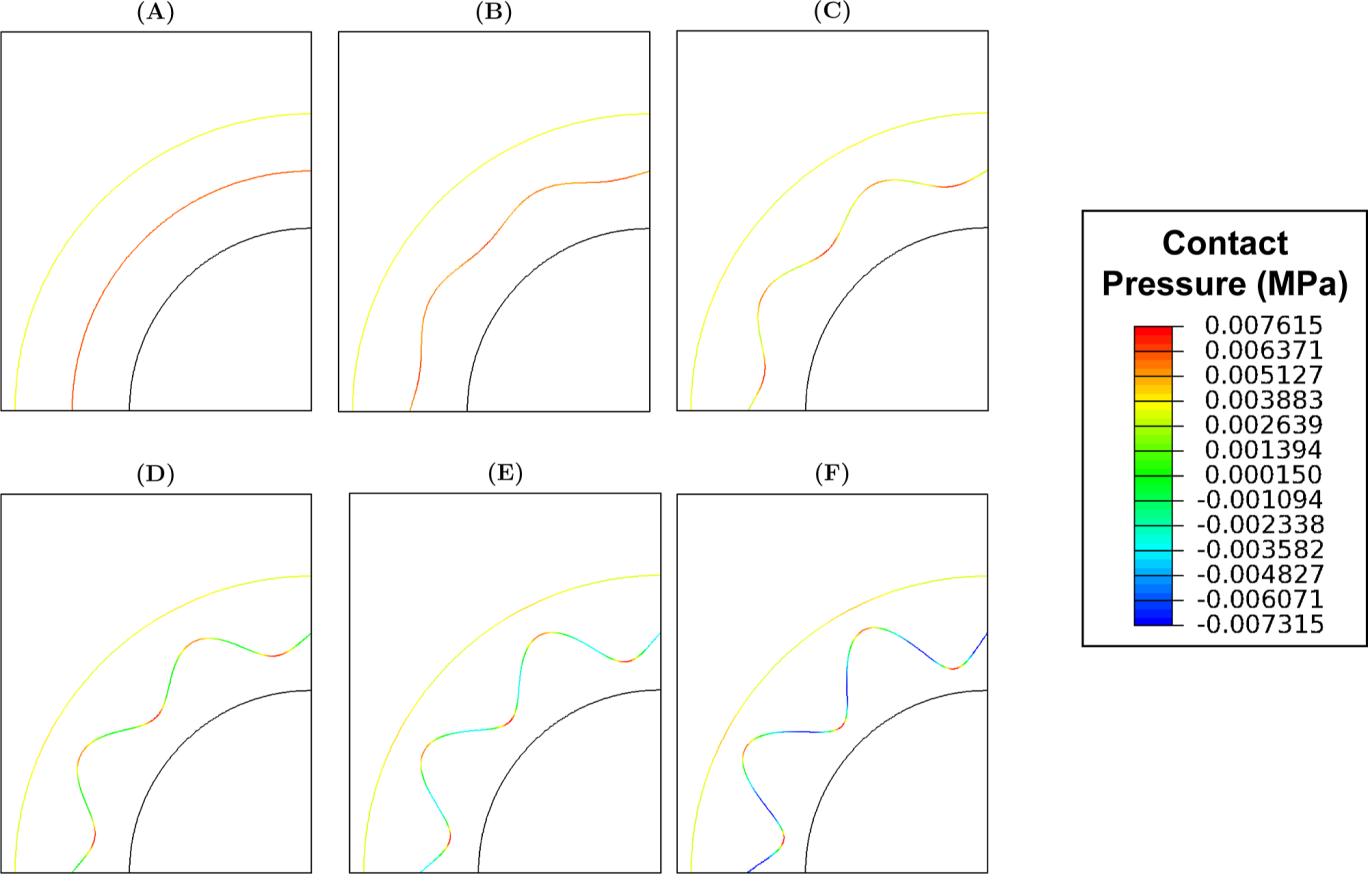
Contact pressures observed at the interface at systolic pressure when (A) A = 0 mm, (B) A = 0.05 mm, (C) A = 0.10 mm, (D) A = 0.15 mm, (E) A = 0.20 mm and (F) A = 0.25 mm.

The variation in σ_θ_ along the interface as a function of ϕ is shown in Figure 9. σ_θ_ was lowest at the peak of the interface wave (ϕ = 90°), where the interface was furthest away from the applied pressure load. On the other hand, σ_θ_ was highest where the interface was closest to the pressure load (ϕ = 270°). The σ_θ_ distribution along the interface became more nonuniform with increasing *A* (Figure 9). For instance, the difference in σ_θ_ at the peak and trough of each wave period increased from 0.016 MPa at *A* = 0.05 mm to 0.060 MPa at *A* = 0.25 mm. The relative decrease and increase in σ_θ_ at ϕ = 90° and ϕ = 270° respectively suggests that, as *A* was increased, the load was shifted from the peaks to the troughs of each wave.

**Figure 9:**
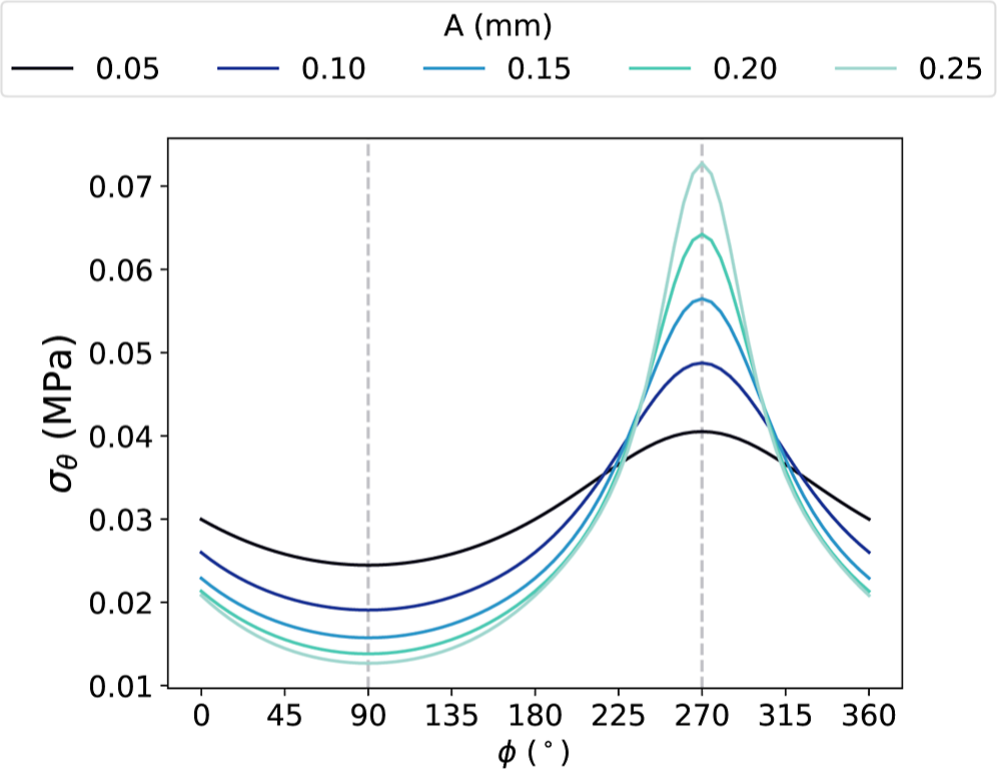
Circumferential stress, σ_θ_, along the interface as a function of phase, ϕ, at systolic pressure. Dashed lines show the locations of minimum and maximum σ_θ_.

#### 4.2.2 Varying Interface Amplitude: Compliance

Increasing the amplitude of the sine wave increased the $\overset{®}{CC}$ of the graft (Figure 10). When *A* = 0 mm, $\overset{®}{CC}$ = 0.0376 mm^2^/mmHg. When *A* = 0.25 mm (the largest amplitude tested in this study), $\overset{®}{CC}$ = 0.0386 ± 0.0002 mm^2^/mmHg. The increase in $\overset{®}{CC}$ observed between consecutive amplitudes (between *A*_0.05_ → *A*_0.10_, *A*_0.10_ → *A*_0.15_, etc) was statistically significant (*P* < 0.001).

**Figure 10:**
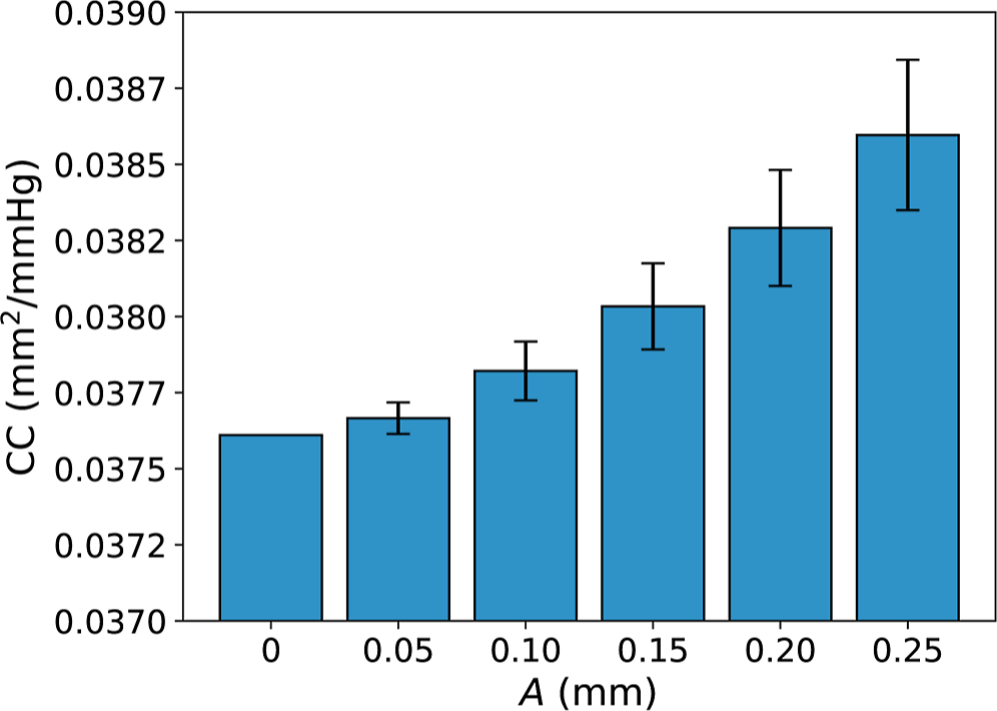
Impact of varying amplitude, *A*, on the mean cross-sectional compliance, $\overset{®}{CC}$ (±standard deviation) of PVA/gelatin grafts.

To investigate the relationship between the spatial composition of the IM and adventitia as a function of *A* and the magnitude of $\overset{®}{CC}$ , sectors corresponding to one complete period of the interdigitated wave were discretized into ten circumferential segments, as detailed in Supporting Information (Section S5). As *A* was increased, the total area of the IM increased slightly (and, subsequently, the total area of the adventitia decreased slightly) due to the curvature of the graft (Table S1). By increasing *A*, circumferential segments closer to the lumen contained more of the compliant outer adventitial layer compared with grafts of lower *A* (Table S2). The reverse trend was observed for the circumferential segments furthest away from the lumen; these segments contained more of the stiff inner IM layer compared with grafts of lower *A*.

Increasing the amplitude of the wave also increased the standard deviation (SD) of the $\overset{®}{CC}$ of the graft (Figure 10). As *A* increased, the radial deformation around the lumen became more nonuniform as a function of ϕ. This was reflected in the larger range of lumen radii (*r*_lumen_) observed at larger amplitudes (Figure 11A). There was a correlation between *r*_lumen_ (and subsequently CC) and the depth of the interface at each ϕ (Figure 11B). *r*_lumen_ was smallest at phases containing more of the inner IM layer, where the interface was furthest away from the lumen (ϕ = 90°). Conversely, *r*_lumen_ was largest at phases containing more of the outer AM layer, where the interface was closest to the lumen (ϕ = 270°).

**Figure 11:**
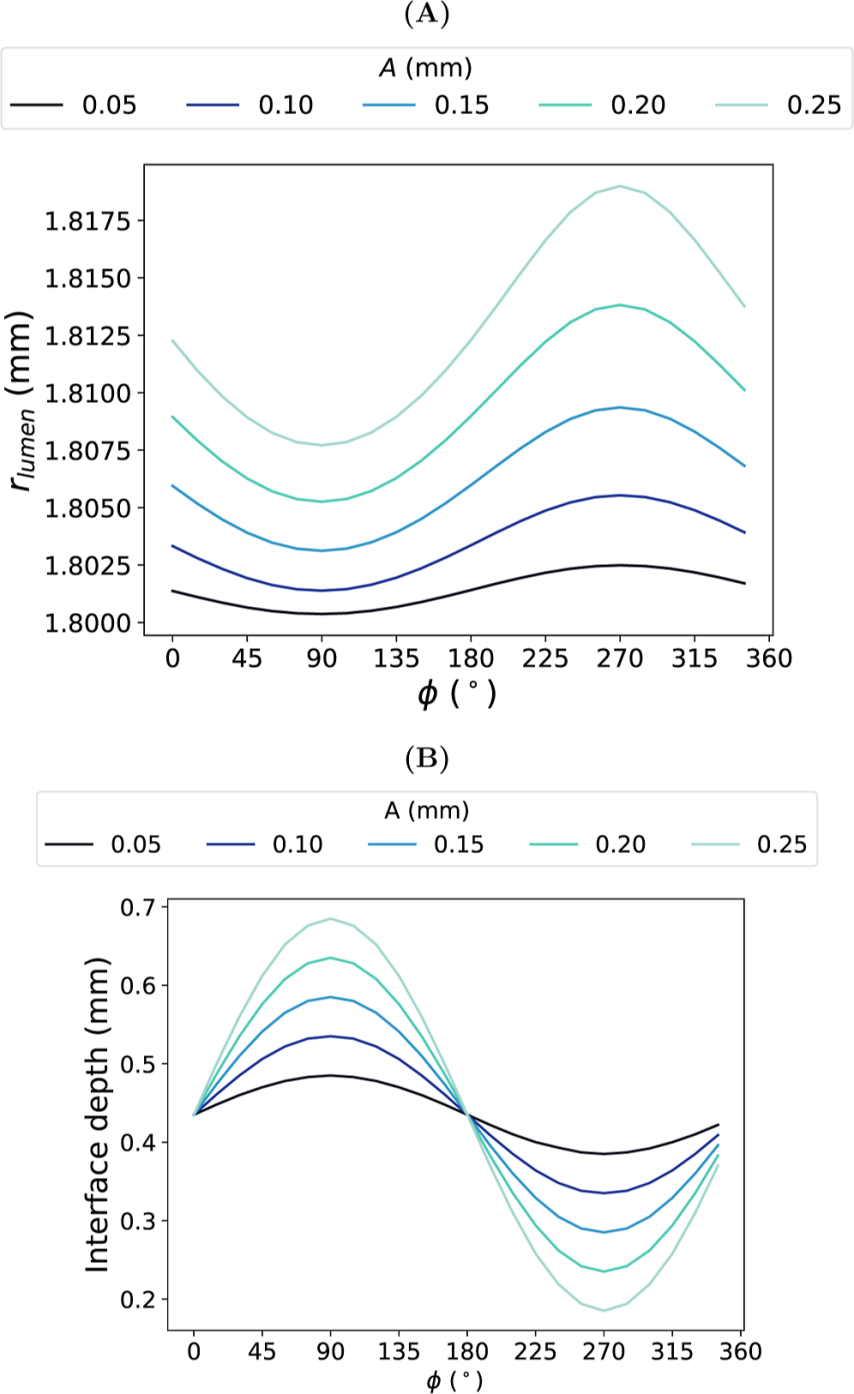
Impact of varying amplitude, *A*, on the (A) lumen radius, *r*_lumen_, at systolic pressure and (B) interface depth of PVA/gelatin grafts. Both (A) and (B) are presented as a function of phase, ϕ.

### 4.3 Impact of Interface Frequency on Graft Biomechanics

#### 4.3.1 Varying Interface Frequency: Stress Distribution

The maximum σ_θ_ of each model was highest at the luminal surface across all phases, irrespective of ω (Figures 12 and 13). However, the magnitude of σ_θ_ at the luminal surface with increasing ω was dependent on ϕ (Figure 13). For instance, at ϕ = 0°, the largest difference in luminal σ_θ_ was observed between the lowest (ω = 6) and highest (ω = 30) frequencies (0.0005 MPa). At ϕ = 270°, the largest difference was observed between ω = 10 and ω = 30 (0.0016 MPa).

**Figure 12:**
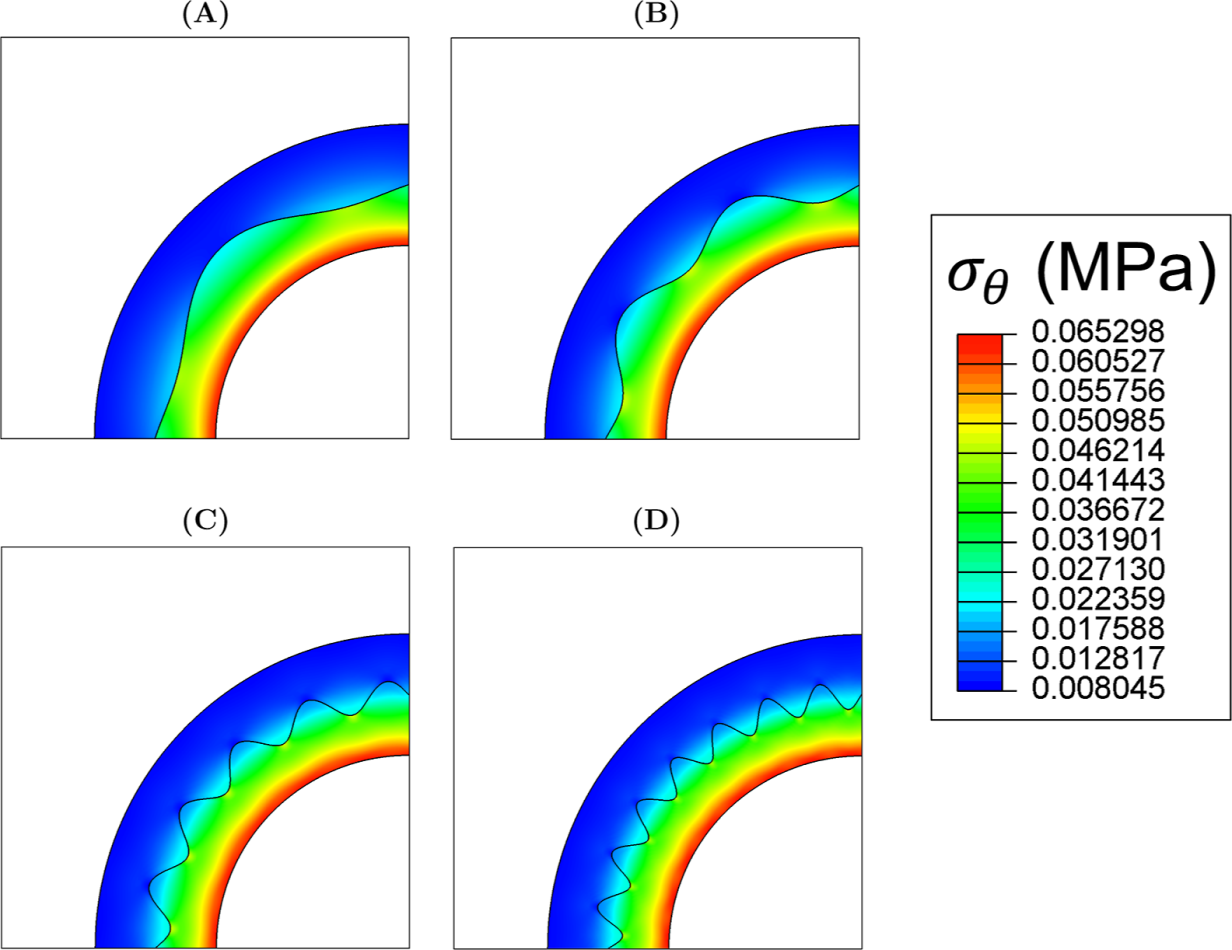
Distribution of circumferential stress, σ_θ_, at systolic pressure when (A) ω = 6, (B) ω = 10, (C) ω = 20 and (D) ω = 30.

**Figure 13:**
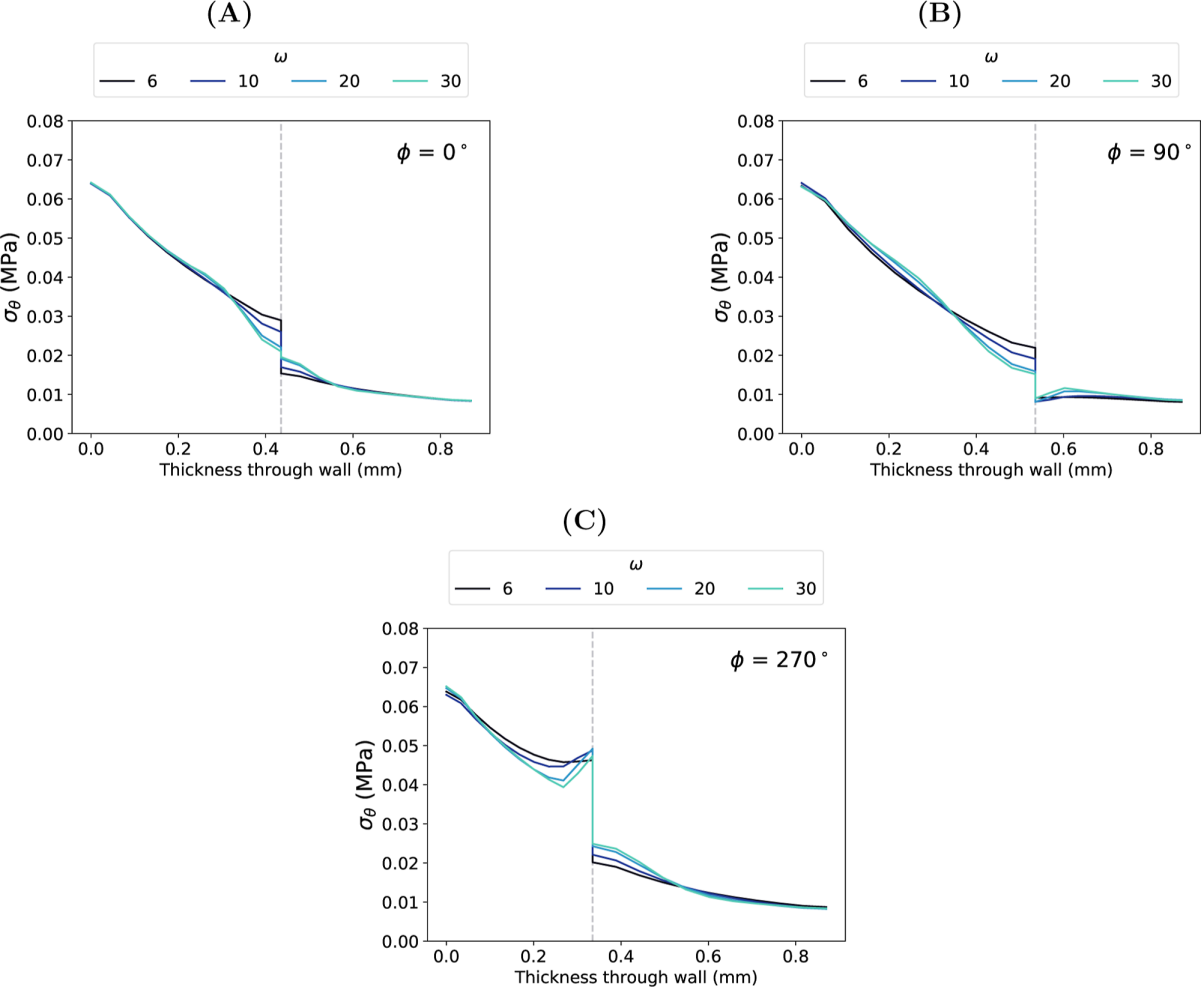
Distribution of transmural circumferential stress, σ_θ_, at systolic pressure with varying frequency, ω, at phase, ϕ, equal to (A) 0°, (B) 90° and (C) 270°. Dashed lines show the interface at each ϕ.

At all values of ϕ, σ_θ_ decreased across the IM-adventitia interface; increasing ω resulted in a smoother transition across the interface (Figure 12). Table 2 reports the decrease in σ_θ_ across the IM-adventitia interface at systolic pressure for the extremes of ϕ (0°, 90° and 270°) and ω (6 and 30).

**Table 2:** Magnitude of the Decrease in Circumferential Stress, σ_θ_, Across the IM-Adventitia Interface at the Extremes of Phase, ϕ (0°, 90° and 270°), and Frequency, ω (6 and 30), at Systolic Pressure

**decrease in σ**_**θ**_ **across the** IM-adventitia **interface**			
**ω**	**ϕ = 0**°	**ϕ = 90**°	**ϕ = 270**°
6	0.0135 MPa	0.0127 MPa	0.0260 MPa
30	0.0014 MPa	0.0062 MPa	0.0225 MPa

When ω = 6 and 10, the highest and lowest interface contact pressures were observed at ϕ = 270° and 0°, respectively (Figure 14). However, when ω = 20 and 30, the highest contact pressure was shifted to ϕ = 90°.

**Figure 14:**
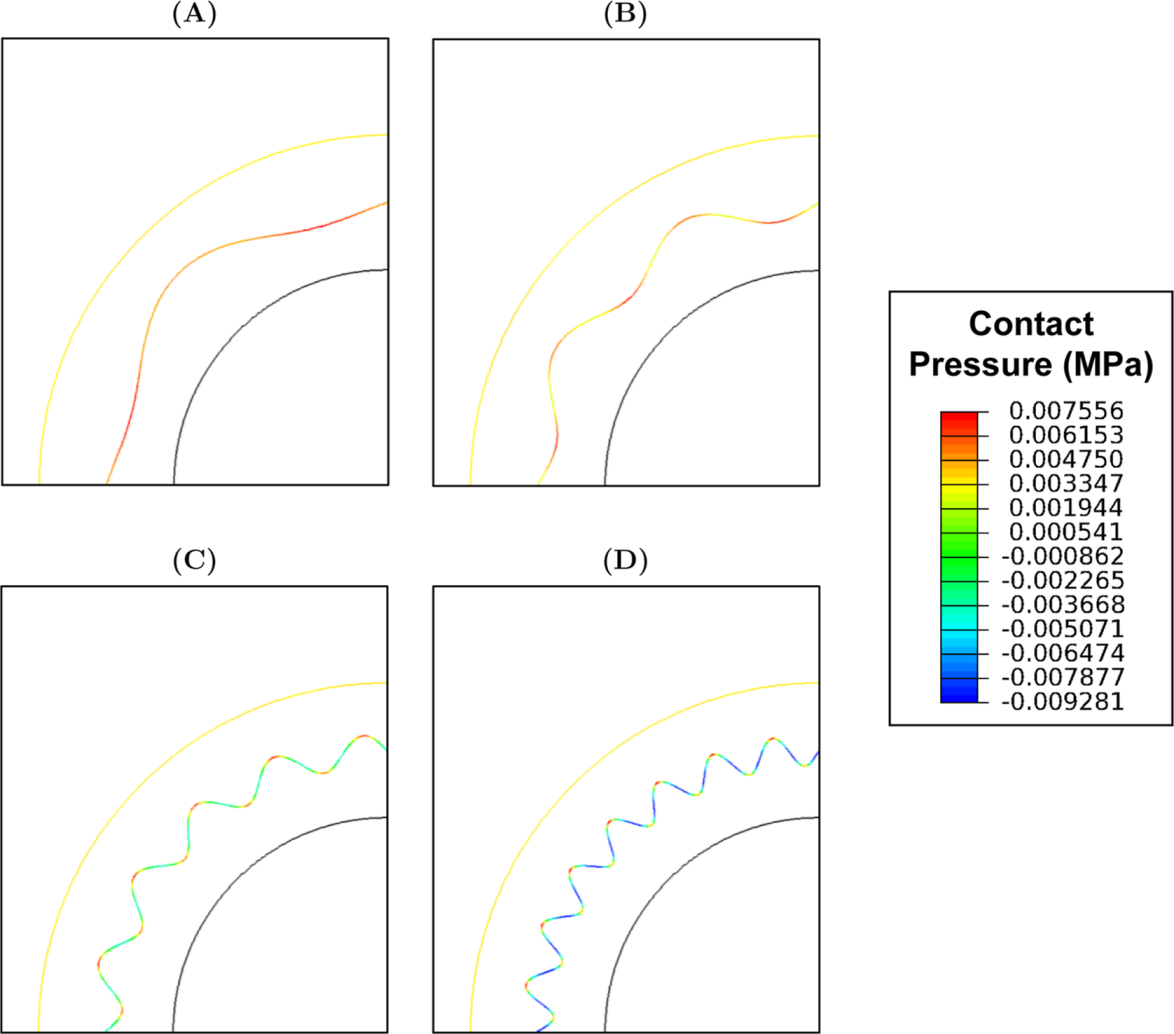
Contact pressures observed at the interface at systolic pressure when (A) ω = 6, (B) ω = 10, (C) ω = 20 and (D) ω = 30.

Figure 15A shows the variation in σ_θ_ along the interface as a function of ϕ for each frequency tested. We observe that σ_θ_ was lowest at ϕ = 90° and highest at ϕ = 270°, reflecting the increased tension observed at the trough of the interface. The difference in σ_θ_ between the peak and trough of each wave increased from 0.024 MPa at ω = 6 to 0.032 MPa at ω = 30.

**Figure 15:**
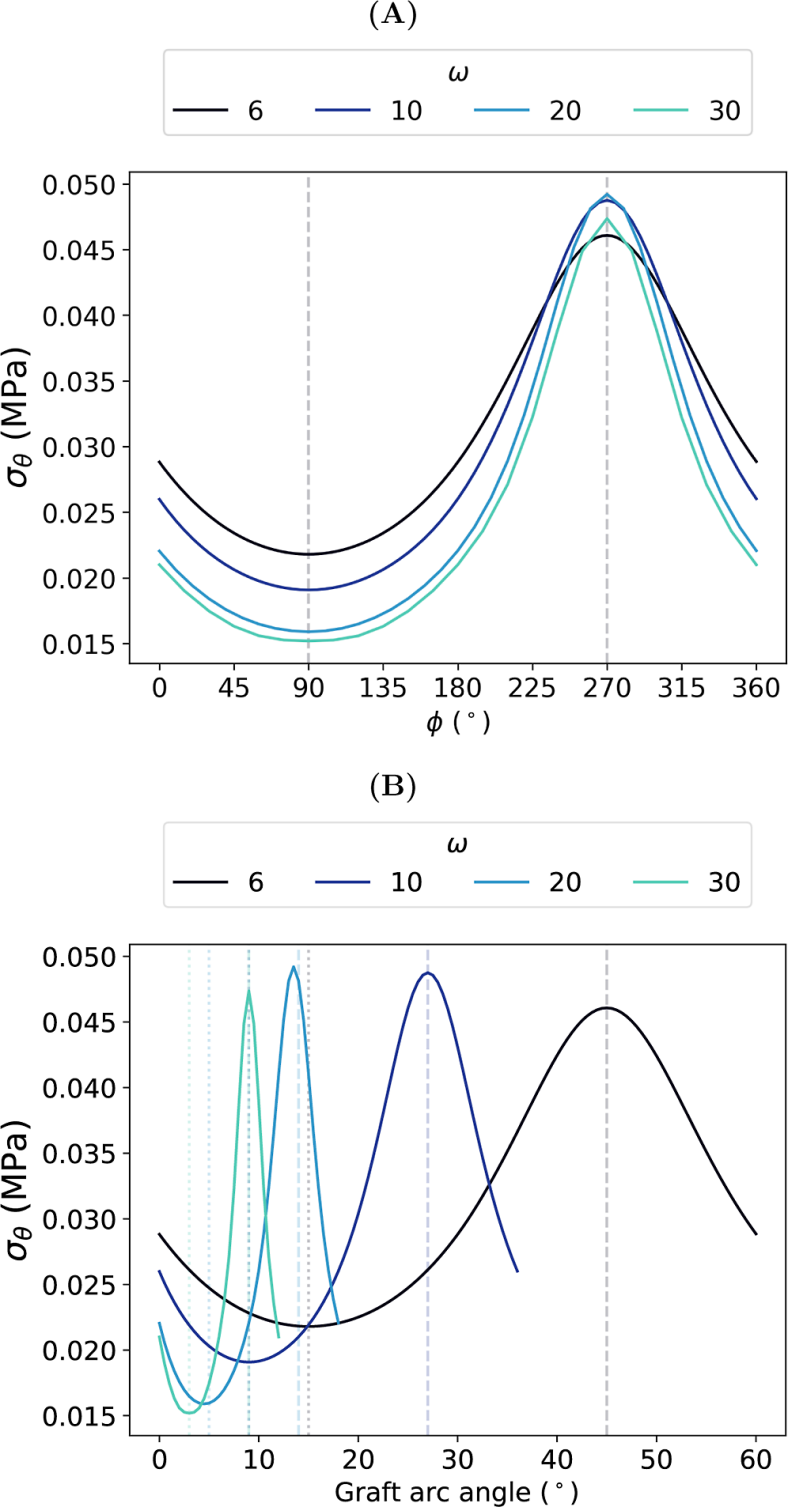
Circumferential stress, σ_θ_, along the interface at systolic pressure as a function of (A) phase, ϕ, of the interface and (B) graft arc angle. Dotted and dashed lines in (B) show arc angles corresponding to ϕ = 90° and ϕ = 270°, respectively.

The circumferential properties of the interface, or the length of the interface around the graft circumference (as determined by the distance covered by one complete wavelength of the interface), varied with ω. Each interface was out of phase with each other; higher frequency interfaces had shorter wavelengths and thus corresponded to shorter arc lengths around the luminal circumference of the graft, as demonstrated graphically in Figure 4. As the arc length was restricted to the absolute dimensions of the graft investigated in this study, Figure 15B instead shows the variation in σ_θ_ along the interface at systolic pressure as a function of graft arc angle.

The interface resisted the intraluminal pressure load along its length. The impact of ω on this phenomenon was 2-fold. First, at high ω, the load acting on one complete wavelength was distributed over a shorter length of interface compared with low ω. Second, the peaks and troughs of the wave became steeper with increasing ω. Figure 15B suggests that the latter increased the stress concentration experienced at the troughs of high ω interfaces. Together, these phenomena may explain the increased difference between the minimum and maximum σ_θ_ values along the interface at higher frequencies, as evidenced by the increased difference in σ_θ_ at the peaks and troughs of each wave.

#### 4.3.2 Varying Interface Frequency: Compliance

Increasing the frequency of the sine wave increased the $\overset{®}{CC}$ of the graft (Figure 16). When ω = 6, $\overset{®}{CC}$ = 0.0378 ± 0.0002 mm^2^/mmHg. When ω = 30, $\overset{®}{CC}$ = 0.0379 ± 9.8 × 10^–6^ mm^2^/mmHg. While the magnitude of $\overset{®}{CC}$ increased with ω, statistical significance was only observed between ω = 6 and ω = 20 (*P* = 0.027) and ω = 6 and ω = 30 (*P* = 0.004).

**Figure 16:**
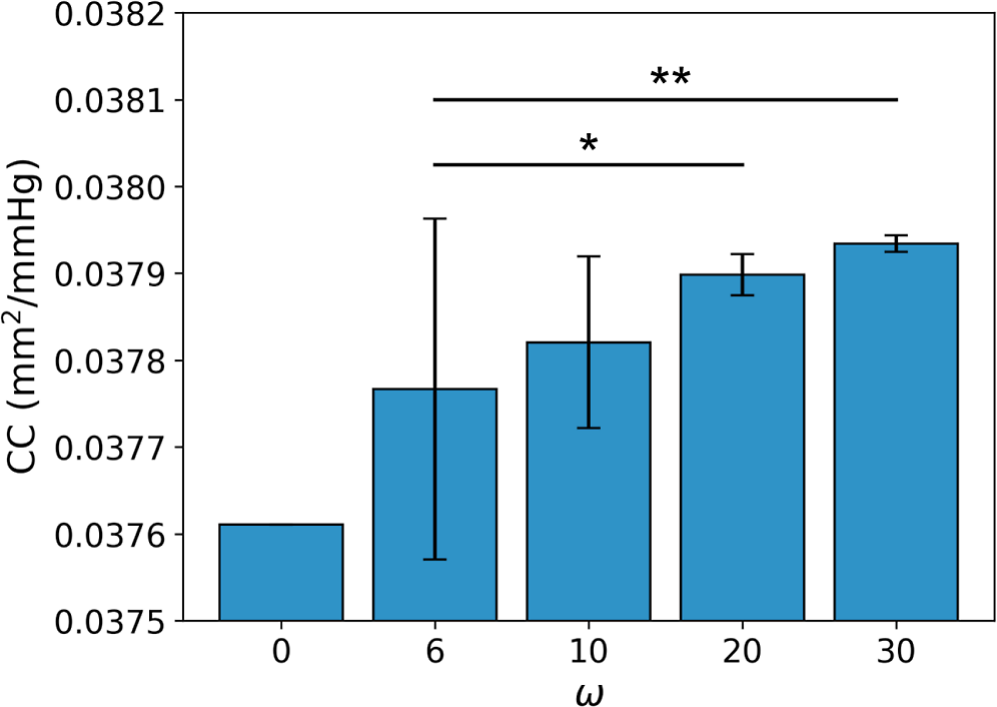
Impact of varying frequency, ω, on the mean cross-sectional compliance, $\overset{®}{CC}$ (±standard deviation) of PVA/gelatin grafts. *p-value, P < 0.05; **p-value, P < 0.01.

The radial deformation became more uniform with increasing ω, as evidenced by the decrease in $\overset{®}{CC}$ SD with ω (Figure 16). This was further illustrated by the decreased range of *r*_lumen_ values at higher frequencies. Figure 17 demonstrates the variation in *r*_lumen_ values between the lowest (ω = 6) and highest (ω = 30) frequencies tested. As the interfaces produced by both waves were out of phase with each other, *r*_lumen_ is plotted as a function of graft arc angle rather than the phase of the interface. Consequently, the peak-to-trough height profile decreased with increasing ω (Supporting Information, Table S4).

**Figure 17:**
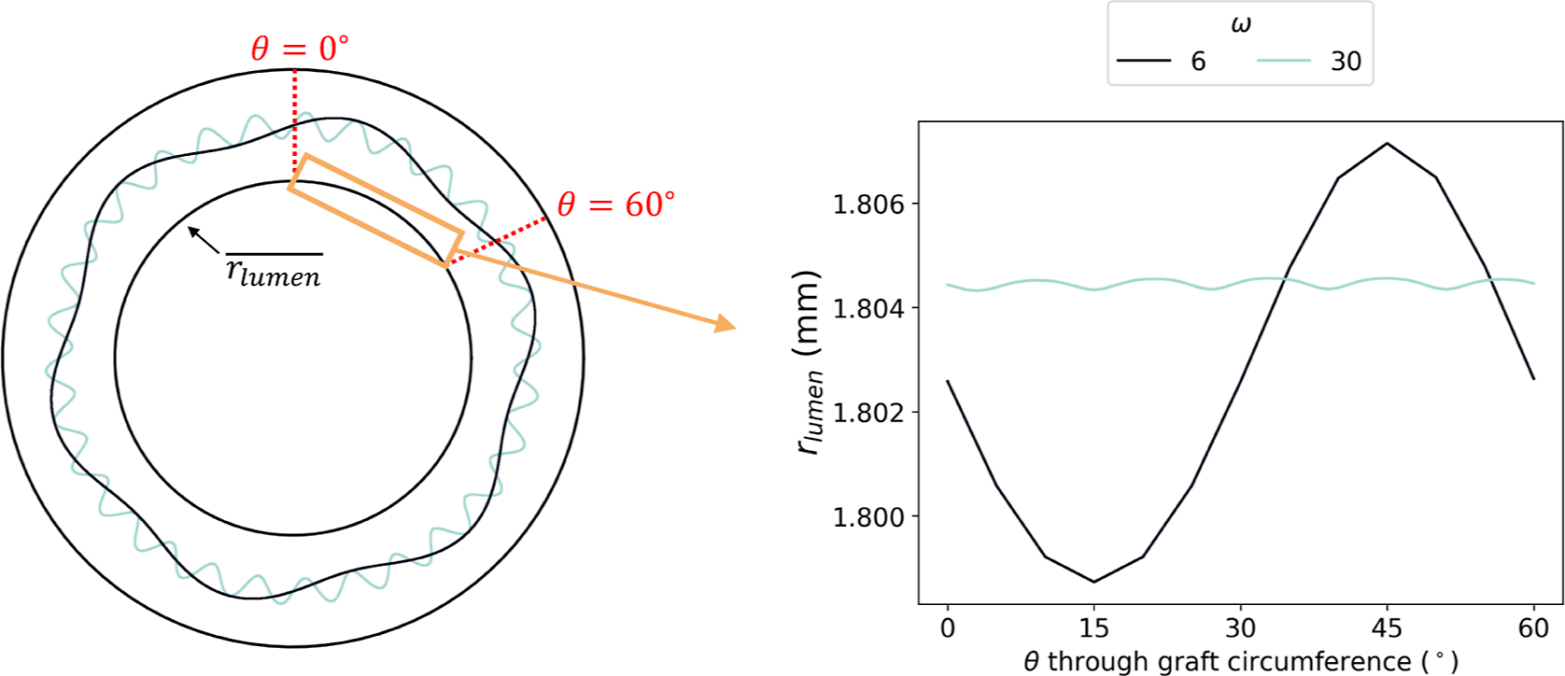
Impact of varying frequency, ω, on lumen radius, *r*_lumen_, at systolic pressure with respect to graft arc angle, θ.

### 4.4 Combining Interface Radius and Amplitude: Compliance

As proof-of-concept for the targeted optimization of graft mechanical behaviors using multiple interface parameters, the combined effect of varying *A* (0.05 mm → 0.25 mm) and *r* (1:2, 1:1 and 2:1) on the $\overset{®}{CC}$ (Figure 18A) and *r*_lumen_ (Figure 18B) of PVA/gelatin grafts was assessed. These values were then compared with literature compliance values for the coronary artery (Table 3). The impact of the interface radius on the stress distribution and compliance is presented in Supporting Information (Sections S3 and S4).

**Figure 18:**
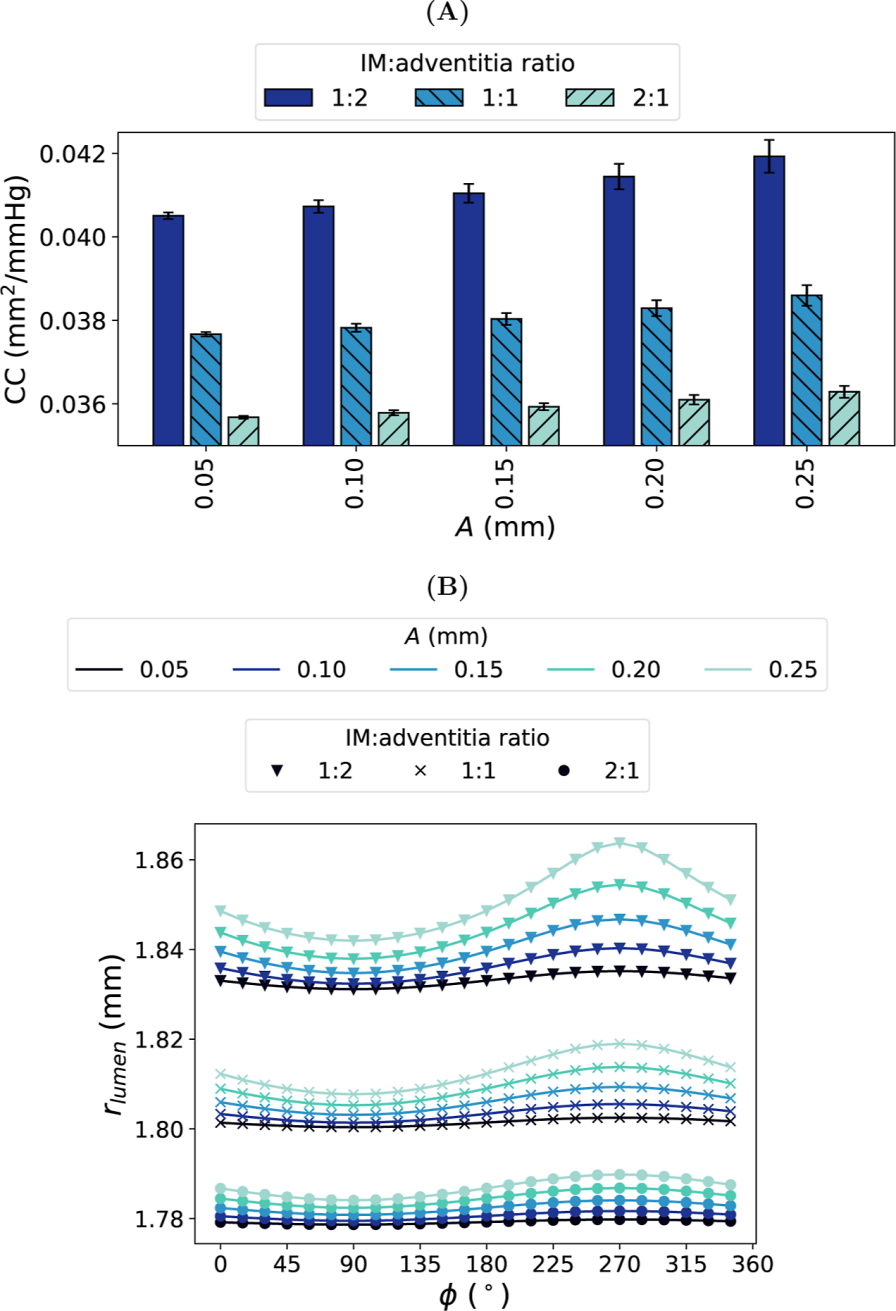
Impact of varying the radius, *r*, and amplitude, *A*, of bilayered interdigitated PVA/gelatin grafts. (A) Mean cross-sectional compliance, $\overset{®}{CC}\pm$ standard deviation; (B) lumen radius, *r*_lumen_, at systolic pressure. In all cases, frequency, ω = 10.

**Table 3:** Compliance of Bilayered Interdigitated PVA/Gelatin Grafts Compared With Human and Porcine Coronary Arteries, Where $\overset{®}{CC}$ Denotes Mean Cross-Sectional Compliance

	**bilayered interdigitated PVA**/**gelatin graft**	**human LAD coronary artery** ^50^	**porcine coronary artery** ^49^
**systolic pressure** (**mmHg**)	120	126.7 ± 5.8	120
**diastolic pressure** (**mmHg**)	80	81.3 ± 3.8	80
$\overset{®}{CC}$ (**mm** ^**2**^/**mmHg**)	0.036–0.042	0.017 ± 3.8	–
$\overset{®}{CC}$ (**%**/**100 mmHg**)	20.12–22.11	–	6.69–8.02

The magnitude of $\overset{®}{CC}$ was dominated by the IM:adventitia ratio (Figure 18A). Decreasing the relative volume of the stiffer IM layer, for instance, incurred a greater increase in $\overset{®}{CC}$ than increasing *A*. This was also reflected in the magnitude of *r*_lumen_; while increasing *A* increased the nonuniformity of *r*_lumen_ with ϕ, Figure 18B shows that the overall magnitude of deformation was primarily governed by the IM:adventitia ratio.

The extent to which larger amplitudes increased the nonuniformity of *r*_lumen_ was reduced by increasing the relative volume of the stiffer IM layer (Figure 18B). Consequently, the lumen surface height profiles were also reduced with increasing IM:A ratio. The smallest height profile was obtained when the IM:adventitia ratio was 2:1 and *A* = 0.05 mm (1.13 μm); conversely, the largest height profile was obtained when the IM:adventitia ratio was 1:2 and *A* = 0.25 mm (21.83 μm). When the IM:adventitia ratio was 1:2, the surface height profile ranged from 4.04 to 21.83 μm. When the volume of the IM was increased such that the IM:adventitia ratio was 2:1, the surface height profile spanned a smaller range of values (1.13–5.75 μm). The full list of peak-to-trough height profiles are listed in Table 4.

**Table 4:** Peak-To-Trough Height Profiles at the Lumen Surface as a Function of Amplitude, *A*, and IM:adventitia Ratio at Systolic Pressure

**PEAK**-**TO**-**TROUGH HEIGHT PROFILE** (**μm**)					
**IM**:**adventitia ratio**	*A* = 0.05 mm	*A* = 0.10 mm	*A* = 0.15 mm	*A* = 0.20 mm	*A* = 0.25 mm
**1:2**	4.04	7.94	12.01	16.56	21.83
**1**:**1**	2.12	4.15	6.24	8.56	11.29
**2:1**	1.13	2.18	3.25	4.42	5.75

Table 3 shows the degree of compliance match between the bilayered interdigitated PVA/gelatin grafts in Figure 18 and those reported for human and porcine coronary arteries. Although variation of *A* and *r* extended the range of compliance values for PVA/gelatin grafts, both the absolute and normalized $\overset{®}{CC}$ remained approximately 100–200% larger compared with the native vessel.

## 5 Discussion

This research aimed to evaulate the mechanical response of biomimetic graft designs, underpinned by the complexity of geometries which may be enabled by AM. This investigation was facilitated by FEA to parametrically assess the impact the variables of the sinusoidal interface (radius, amplitude and frequency). Prior to the main findings of the research, the study assessed the suitability of the computational approach to model the contact behavior between PVA/gelatin graft interfaces.

### 5.1 Modeling the Contact Between Interdigitated Surfaces

A lack of literature defining the contact interactions between two PVA/gelatin surfaces and the importance of accurately defining such interactions when increasing graft design complexity, was highlighted. Thus, before analyzing the impact of a complex interface on transmural stress distribution, it was necessary to establish a suitable contact formulation that would emulate the expected movement between graft layers without introducing unrealistic displacements and/or stress concentrations at the interface.

Figure S2 demonstrates the stress independency of laminated models when using friction coefficients, implementing rough friction or tying the surfaces together. σ_θ_ was governed only by the relative material properties and thicknesses of both layers. These data verified assumptions made whereby, in lieu of a friction coefficient between two PVA cryogel surfaces, a friction coefficient of 0.04 was chosen according to the friction coefficient of PVA cryogel and titanium alloy.^45^ However, the same friction coefficient caused antiparallel sliding of the two layers upon introduction of the interdigitated interface. This tangential slip amplified stress concentrations at the peaks and troughs of the interdigitated wave, regardless of the PVA/gelatin composition used to model each layer.

The adhesive behavior between interfacing cryogel layers under an applied load (and the magnitude of force responsible for failure) is not well-defined for any mode of loading in the literature. Instead, this behavior must be qualitatively inferred from existing studies of hydrogel-based FGBMs. This lack of knowledge is further narrowed by the choice of manufacturing method used to fabricate heterogeneous grafts. For instance, the casting method proposed by Wahab et al. exploited the diffusion between adjacent PVA solutions to form continuously graded constructs with intermediary zones.^31^ In this case, tying adjacent interfaces may be an applicable assumption. Conversely, materials printed using extrusion-based AM techniques are subject to interface formation both between and within successive print layers.^13,51^ When PVA is extruded onto a frozen print bed, physical cross-links form between adjacent filaments.^13^ However, the extent of cross-linking is governed by the freezing process. Interfacing regions may be vulnerable to excessive deformation and delamination when placed under tension;^51^ the decreased bond strength between parallel strands of 3D-printed PVA cryogel has been recognized as a potential mechanism of premature sample failure under uniaxial tension.^13^ Tied surface constraints likely oversimplify the local deformations and displacements that may occur between adjacent printed regions under physiological loading; on the other hand, static friction coefficients are unable to model the expected delamination failure mechanism.

Caution should therefore be exercised when using friction coefficients to model the contact between interdigitated or interlocking hydrogel layers. For the purpose of this study, a rough (infinite) friction constraint was implemented to prohibit relative slip between the two graft layers while allowing analysis of the contact pressure at the interface. This simplification assumes the two layers remain adhered when the graft is pressurized to systolic pressure. To validate this assumption, experimental data quantifying the local displacement of interdigitated interfaces under physiologically relevant loading (for example, tracking local strains through digital image correlation^51^) are needed.

The conclusions derived from these simulations further reinforce the need to adequately report and/or explore the contact between interfacing layers in the FEA of multilayered vascular grafts. In a study of a composite vascular scaffold comprised of a knitted wire mesh embedded in a polyurethane scaffold, Sirry et al. used static friction coefficients between 0.2 and 0.55 to model the tangential surface interactions between the two components.^52^ Meanwhile, Byrne et al. tied separate material sections of their fiber-reinforced graft using a tie constraint.^16^ Though both studies developed grafts using alternative VMMs, the inclusion of complex geometrical elements—and thus more complex stress–strain behavior—warrants further exploration of contact properties of VMMs in interfacing regions.

### 5.2 Analysis of Sine Wave Parameters on Graft Biomechanics

Sinusoidal waveforms are attractive design components in cardiovascular tissue engineering as they mimic the crimping (and subsequent stiffening under increasing pressure) behavior exhibited by collagen fibers^41^ and elastic lamellae. For instance, Byrne et al. embedded sinusoidal polyurethane fibers in a soft silicone matrix to provide hyperelastic reinforcement to composite medium-large vascular grafts.^16^ Sinusoidal filament networks have also been explored in the wider context of soft tissue engineering; by varying the filament period and amplitude-to-period ratio, Meng et al. demonstrated how the mechanical properties of polycaprolactone scaffolds can be tuned between 27 and 1944 kPa.^53^ Importantly, the ability to create mathematically driven toolpaths using AM enables printing of sinusoidal PVA/gelatin cryogel fibers using subzero AM.^41^

A bioinspired sinusoid was therefore explored as the complex interface in this study. Subsequently, it was determined that the transmural σ_θ_ distribution, luminal displacement and CC of bilayered interdigitated PVA/gelatin grafts subjected to physiological pressures were dependent on the phase and/or graft angle at which they were measured. This was an important finding as it emphasized the need to evaluate stress patterns (including concentrations) and lumen displacement with respect to the interface waveform.

Compared with noninterdigitated interfaces (Supporting Information, Section S2), the transmural σ_θ_ gradients at ϕ = 0° and ϕ = 180° (which have the same radial depth as the noninterdigitated interface) were ‘phased’, i.e., the stress distribution was more continuous. Moreover, the degree of phasing increased with increasing *A*. This behavior mimics the role of residual stresses in native coronary arteries, which minimize intramural σ_θ_ gradients derived from blood pressure.^54^ However, this phased behavior was achieved at the expense of increased σ_θ_ at the luminal surface (which residual stresses have been found to decrease^55^) and intramural σ_θ_ concentrations at ϕ = 270°, both of which were exacerbated at higher amplitudes.

Troughs are an intrinsic property of sinusoidal waveforms. High intramural stress concentrations alter endothelial cell (EC) morphology and are associated with decreased turnover of extracellular matrix proteins.^56,57^ Furthermore, based on studies of coronary arteries,^58,59^ it is speculated that increased stretching at the troughs of the interface may increase EC permeability and facilitate low-density lipoproteins accumulation in the IM layer of the graft. Together, these data suggest that sinusoidal interfaces may provide both atheroprotective and atherogenic cues to the endothelium. Experimental validation of these graft designs should therefore aim to investigate the impact of interface variables on EC expression of cyclic stretch-mediated mechanosensitive genes.

Intriguingly, the phased behavior at ϕ = 0°—as yielded at higher amplitudes—was achievable at lower amplitudes by increasing ω. This was achieved without significantly increasing σ_θ_ at the luminal surface; moreover, as *A* was kept constant for each frequency simulation, σ_θ_ concentrations at ϕ = 270° were comparatively lower than at higher *A*. It is hypothesized that the phased behavior of σ_θ_ with increasing *A* and ω are caused by the bifurcation of behavior as a direct consequence of the interface. Bifurcation theory is a deeply mathematical concept that describes the way that a system varies under parametric changes.^60^ In the context of this research, it could explain how σ_θ_ behaves relative to the interface until a threshold *A* and ω is reached, upon which is acts independently. For instance, consider the σ_θ_ contour maps in Figure 12: when ω = 6, the distribution of σ_θ_ was dependent on the interface geometry; when ω = 20, it became independent of the interface geometry. This behavior may be attributed to an assumption related to the FE model (for example, mesh density) or may be representative of physical phenomena. Nevertheless, it is hypothesized that the combination of *A* and ω may be tuned to maximize atheroprotective σ_θ_-derived cues. Future work should therefore look to evaluate a combined amplitude/frequency parameter sweep on transmural σ_θ_ patterns using both in silico and in vitro testing.

In addition to mediating the σ_θ_ distribution, the magnitude and range of *CC* increased with increasing *r*, *A* and ω. Of the three sinusoidal parameters, *r* and *A* had the biggest impact on the magnitude of $\overset{®}{CC}$ . This was unsurprising given the reliance of lumen deformation and compliance on radial composition. Increasing *r* increased the relative amount of stiff IM layer, decreasing CC. As *A* was increased, the relative amount of compliant outer adventitial layer in circumferential segments closer to the lumen increased. Thus, in grafts with larger *A*, the compliant adventitia bore more of the pressure load compared with grafts of lower *A*. This resulted in larger $\overset{®}{CC}$ as a function of *A*, as shown in Figure 10. This trend is in agreement with the study by Byrne et al., where grafts designed with the largest fiber amplitude yielded the highest compliance across mean pressures of 0–130 mmHg.^16^

Furthermore, as *A* was increased, the radial deformation around the lumen became more nonuniform as a function of ϕ. This was reflected in the larger range of lumen radii observed at larger amplitudes (Figure 11A). *r*_lumen_ was smallest at phases containing more of the inner IM layer, where the interface was furthest away from the lumen (ϕ = 90°). The increased stiffness at these phases resulted in decreased radial deformation, yielding lower CC values. On the other hand, *r*_lumen_ was largest at phases containing more of the outer adventitial layer (ϕ = 90°). The decreased stiffness at these phases resulted in increased radial deformation and larger CC values.

### 5.3 Study Reflections

An interesting consequence of the interdigitated interface was the introduction of pressure-induced luminal surface waviness due to nonuniform radial expansion when loaded to systole. If a VMM is to be used in endothelium-contacting and blood-interfacing graft constructs, it is imperative to understand the impact that its surface topography has on its hemocompatibility. For instance, VMMs with microscale surface roughness have higher thromobogenic potential than those with nanoscale roughness due to the increased surface area for platelet activation and adhesion.^61^ On the other hand, incorporating submicron ridges into the VMM surface (0.05–2 μm) may attenuate thromobogenicity by limiting platelet adhesion to the peaks of the grooves, where the surface area is minimized.^61,62^ Furthermore, compared with smooth surfaces, increased endothelialisation rates and EC alignment have been observed on VMMs with structured anisotropic nano- and microscale ridged topographies.^63,64^ It must be emphasized that the in silico expansion behavior of the interdigitated grafts reported in this study did not consider the initial surface roughness and require experimental validation. However, the results presented suggest that interface design may be a viable target toward the generation of surface topographies that are representative of the native intima under physiological loading.

For each graft design, the peak-to-trough height of the lumen at systolic pressure was reported to quantify the maximum deviation in the lumen height profile. Of the three sinusoidal interface parameters explored in this study, the luminal height profile was most susceptible to variations in amplitude and radius; the largest height profile (21.83 μm) was obtained for the largest amplitude when the IM:adventitia ratio was set to 1:2. The surface topographies of the grafts in this study met the standard reported for porcine left anterior descending (LAD) coronary arteries, where circumferential and longitudinal height profiles span approximately 10 μm.^65,66^ While submicron features elicit desirable surface characteristics compared with smooth surfaces, there is a growing body of evidence associating increased intimal surface roughness with the onset and progression of atherosclerosis.^67^ Computational fluid dynamics models have indicated that local minima in the surface height profile of porcine LAD coronary arteries correspond to regions of atherogenic wall shear stress.^66^ As the sinusoidal interfaces formed sinusoidal surface features, these findings suggest scope for research into alternative interface waveforms that promote hemocompatible topographies while minimizing atherogenic trough formation.

It is evident that the $\overset{®}{CC}$ of the PVA/gelatin graft designs evaluated in this study were more compliant between diastolic and systolic pressure than the native coronary artery wall. Given that the interdigitated waveform increases compliance relative to a noninterdigitated graft, this research further highlights the need for cryogel compositions with sufficient strain-stiffening behavior over physiological pressure. To achieve a closer compliance match in terms of magnitude, stiffer compositions of PVA/gelatin cryogel are required.

The ability to control graft compliance through interface design has exciting consequences for the development of coronary artery tissue replacements. Synthetic grafts are often developed according to an idealized isotropic or orthotropic tubular geometry, yet coronary arteries exhibit location-specific compliance along their length.^49^ Extending the parametrically driven toolpath developed in this study into a third dimension could allow for graded compliance along the length of the graft. Graded compliance could also help to reduce compliance mismatch at anastomoses or even mediate disrupted flow patterns experienced near bifurcations.^68,69^

## 6 Conclusion

This study hypothesized that a sinusoidal interface could be used to alter the stress pattern across interfacing tissue layers and induce gradual changes in transmural stress—a physiologically relevant, yet critically underexplored, concept in the field of cardiovascular tissue engineering that has the potential to reduce atherogenic stress concentrations linked to graft failure. Drawing on the complex geometries enabled by AM, this research used FEA to explore the parametric, impact of the design variables (radius, amplitude and frequency) on graft biomechanics. Compared to the laminated models, it was found that the inclusion of an interdigitated interface ‘phased’ the transmural σ_θ_ to decrease the discontinuity of stress across the interface. This behavior was observed with increasing *A* and ω and was dependent upon the phase of the interface wave or the graft arc angle. This research demonstrates that incorporating a sinusoidal interface into soft tissue grafts, such as arterial, could enable the design of a functionally graded transmural stress distribution as a function of *A* and ω. Furthermore, the design and computational approach presented in this research may be translated more widely to the design of synthetic, biomimetic grafts.

## Supplementary Material



## Notes

Supporting Information Additional experimental details and preliminary results (PDF)
