# Supplementary material for: Simulating the Transmural Mechanical Response of Functionally Graded Arterial Grafts
Source: ACS Appl Bio Mater. 2025 Nov 15;8(12):10800–17. doi: 10.1021/acsabm.5c01506 (PMC12709613; doi:10.1021/acsabm.5c01506)
Supplement: Supplementary file 1 [file mt5c01506_si_001.pdf]

Simulating the Transmural Mechanical Response of Functionally Graded Arterial Grafts

Supplementary Data

Katie L. Fegan[1,2], Amy V. Tansell *[3], Asif J. Iqbal[4], and Lauren E.J. Thomas-Seale[1]

[1]*Physical Sciences for Health Centre for Doctoral Training, University of Birmingham, Birmingham, UK*
[2]*Department of Mechanical Engineering, School of Engineering, University of Birmingham, Birmingham, UK*
[3]*School of Mathematics, University of Birmingham, Birmingham, UK*
[4]*Department of Cardiovascular Sciences, University of Birmingham, Birmingham, UK*

S1 Discretisation Analysis

The two radii of the sector were chosen such that the sector contained one complete wave period: $r_1 = \phi = 0°$; and $r_2 = \phi = 360°$. The Cartesian coordinates of the nodes corresponding to the outer boundaries of each sector were extracted from their respective Abaqus model and imported into Fusion 360.

The circumferential variation in IM and adventitial layers was measured through circumferential discretisation of each sector into ten segments of equal depth (Figure S1). For each segment, the areas of the IM and adventitia were measured in Fusion 360 and converted into IM:adventitia ratios using

$$\text{IM:adventitia} = \frac{[\text{Area of IM}]}{[\text{Area of segment}]} : \frac{[\text{Area of adventitia}]}{[\text{Area of segment}]}. \tag{1}$$

*Corresponding Author: AXT673@student.bham.ac.uk

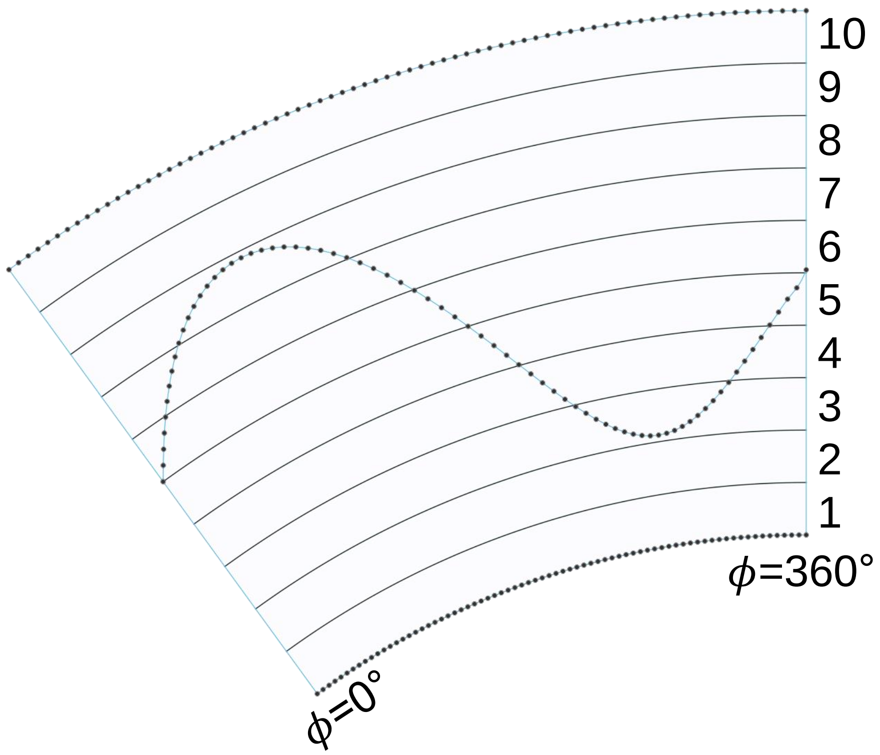

Figure S1: Example reconstruction of a graft sector in Fusion 360, shown for an interdigitated graft where $A = 0.25$ mm, $\omega = 10$ and $r = 1.815$ mm. Circumferential segments are numbered from 1–10.

S2 Modelling the Contact Between Interdigitated Surfaces

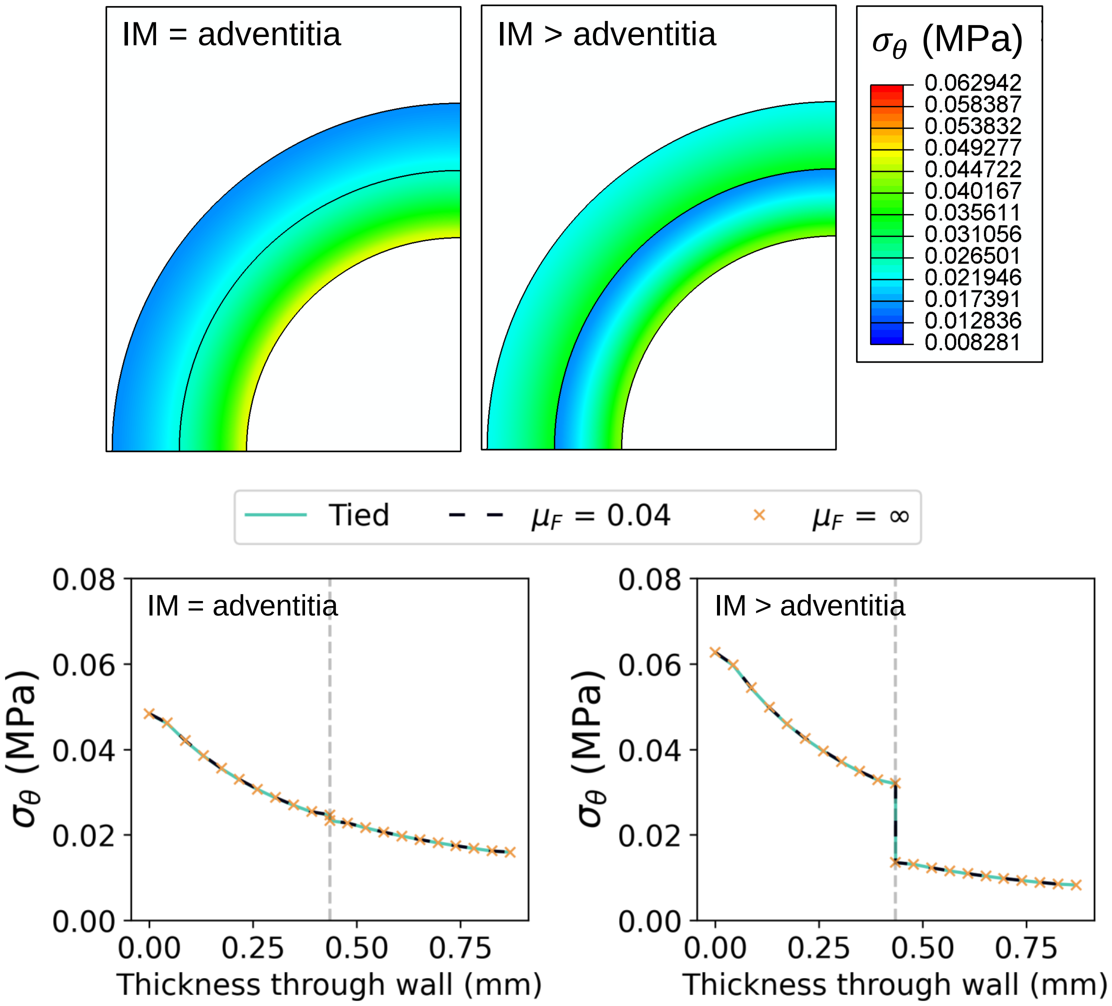

Figure S2: Comparison of friction, rough friction and tied contact formulations on a bi-layered non-interdigitated graft, where the IM and adventitia were modelled using the same PVA/gelatin composition (IM = adventitia; P_9G_1-A-C) and different PVA/gelatin compositions (IM > adventitia; IM = P_9G_1-A-C; adventitia = P_9G_1-B-NC).

As a control experiment, Figure S2 shows the impact of contact formulation on the transmural σ_θ distribution of bi-layered non-interdigitated grafts ($A = 0$ and $\omega = 0$). No difference in σ_θ distribution was observed between friction, rough friction and tied contact models; the resulting stress pattern was independent of contact formulation and was instead determined by the overall dimensions of the graft and the material properties of each layer, with heterogeneous material properties amplifying the discontinuity at the interface.

Different σ_θ distributions were observed between contact formulations for the interdigitated model. These differences were observed irrespective of the relative material properties of the IM and adventitia; Figure S3 shows the σ_θ distribution where the IM and adventitia layers were modelled using the same PVA/gelatin composition and Figure S4 shows where the IM was stiffer than the adventitia. For all contact formulations, σ_θ concentrations were observed in both the IM and adventitia at the peaks ($\phi = 90°$) and troughs ($\phi = 270°$) of the interdigitated wave. Using the rough friction coefficient and tying the two surfaces together yielded equivalent σ_θ distribution patterns. However, the magnitude of the stress concentrations were amplified in the friction coefficient model. The σ_θ at the interface at $\phi = 270°$ increased from 0.056 MPa in the rough friction model and 0.055 MPa in the tied surface model to 0.188 MPa in the friction model (Figure S4).

The amplified stress concentrations observed at the interface of the friction model were accompanied by increased σ_θ discontinuities across the IM-adventitia interface. Using $\phi = 270°$ as the example once more, σ_θ decreases across the IM-adventitia interface by 0.030 MPa, 0.028 MPa and 0.195 MPa in the rough friction, tied surface and friction models, respectively (Figure S4).

Analysis of the tangential displacement (x_θ) of the IM and adventitia showed the relative movement of the interface under the applied pressure load (Figure S5). In the rough friction and tied surface models, tangential slip was constrained. At all phases, the x_θ of the IM and adventitia layers occurred in parallel directions (Figure S5A). The direction of x_θ between $\phi = 90$–$270°$ and $\phi = 270$–$90°$ indicate stretching of the interface under loading. These displacements corresponded to regions of high tensile stress in the graft: in the IM layer, for instance, tensile stress concentrations were observed at the troughs ($\phi = 270°$) of the wave (Figure S5B). Simultaneously, tensile stress concentrations were observed in the peaks of the adventitia ($\phi = 90°$).

Meanwhile, tangential slip in the 0.04 friction coefficient model caused the IM and adventitia to slip against one another. This was evidenced by the opposing displacement direction (x_θ) of the IM and adventitia at the interface (Figure S5C). While high tensile stress (stretching) was still observed at the troughs of the IM layer, compressive stress concentrations were introduced in the peaks of the adventitia (Figure S5D). The absolute magnitude of x_θ for the friction coefficient model was also greater (0.038 mm) compared with the rough friction (0.011 mm) and tied (0.010 mm) models (Figures S5C and S6)

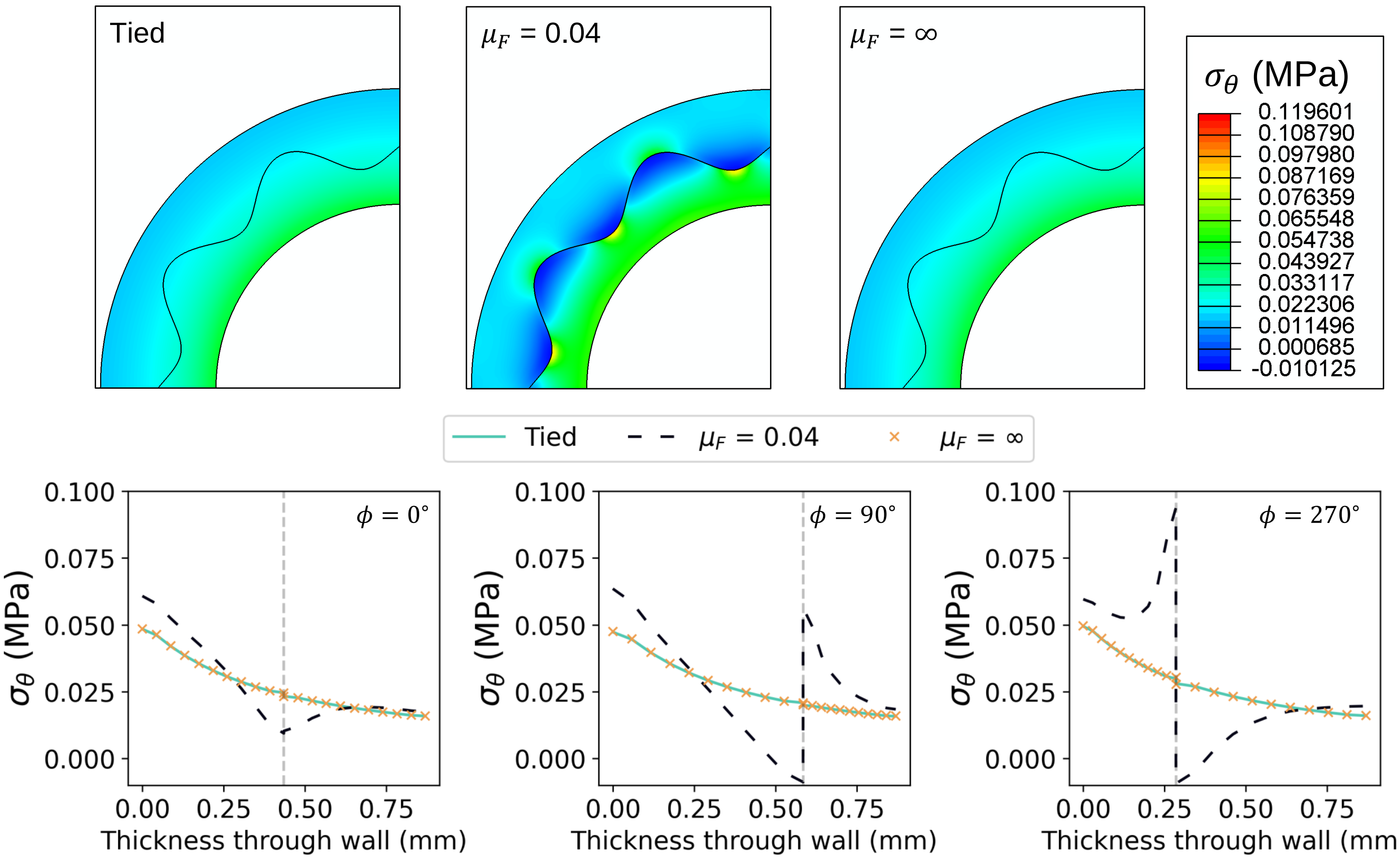

Figure S3: σ_θ distributions for three contact formulations (tied / $\mu_F = 0.04$ / $\mu_F = \infty$) when the IM and adventitia were modelled using the same PVA/gelatin composition (P_9G_1-A-C). Layer boundaries are represented by grey dashed lines.

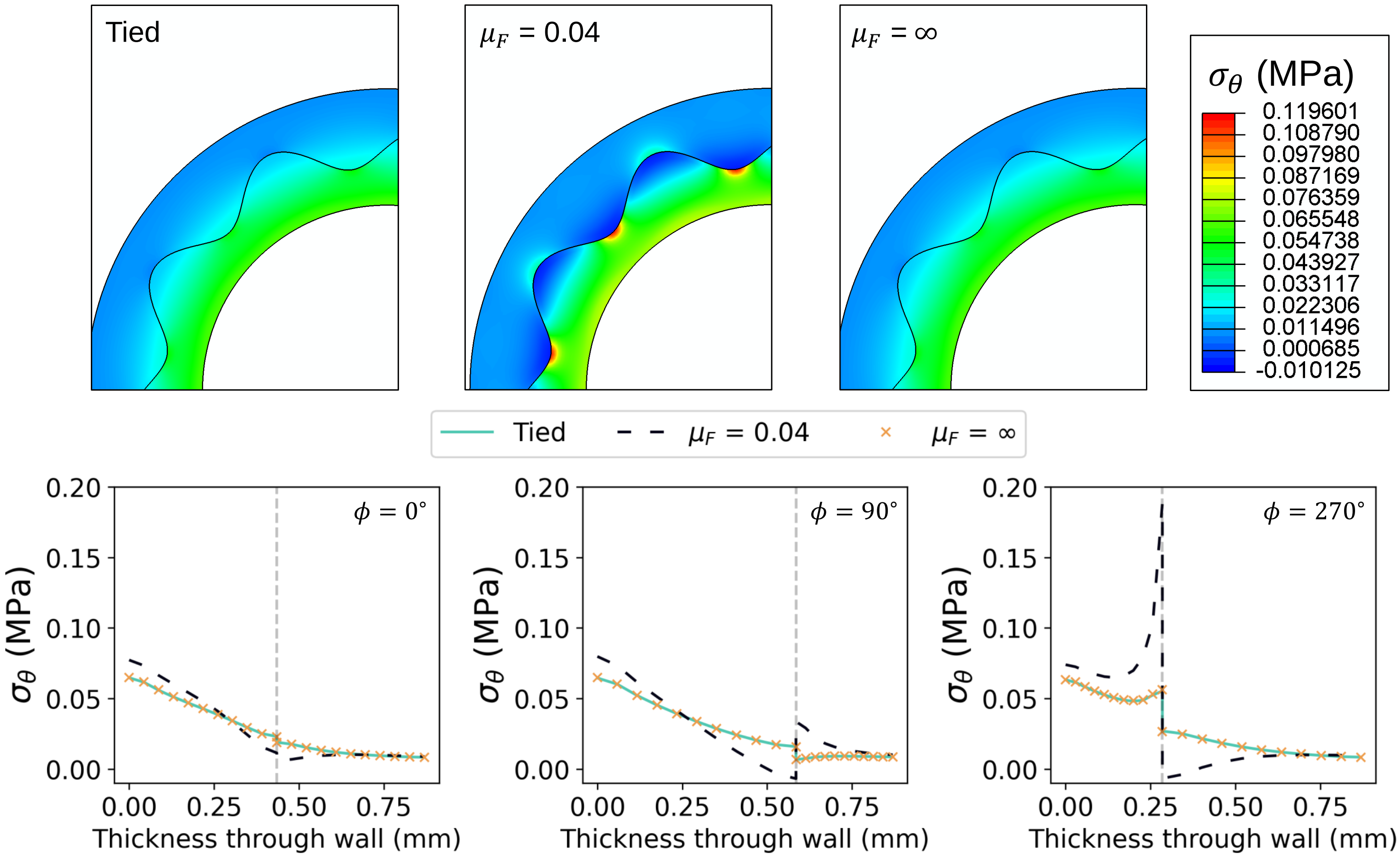

Figure S4: σ_θ distributions for three contact formulations (tied / $\mu_F = 0.04$ / $\mu_F = \infty$) when the IM (P_9G_1-A-C) was stiffer than the adventitia (P_9G_1-B-NC). Layer boundaries are represented by grey dashed lines.

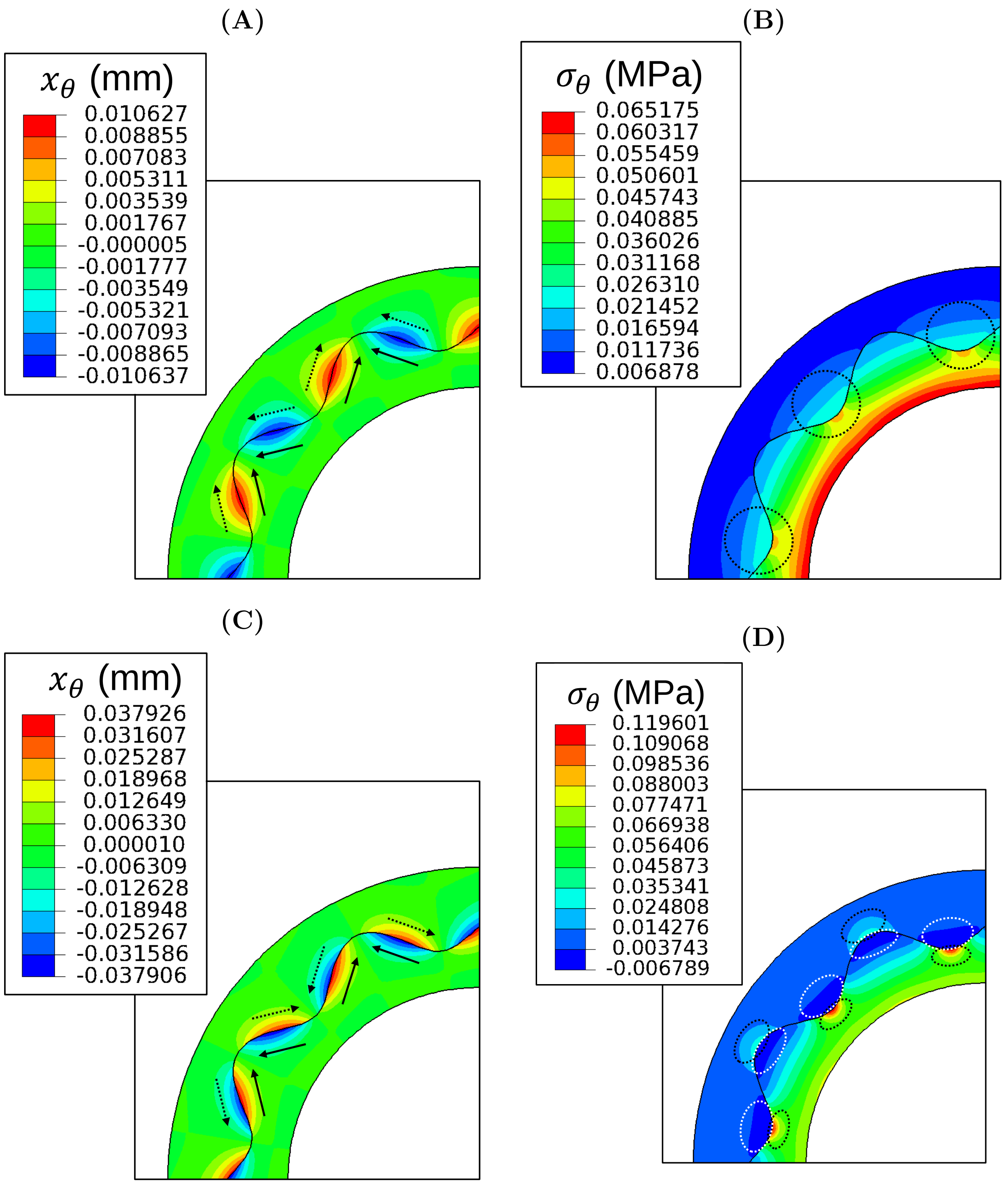

Figure S5: **(A)** x_θ and **(B)** σ_θ of the rough friction coefficient model; **(C)** x_θ and **(D)** σ_θ of the 0.04 friction coefficient model (IM = P_9G_1-A-C, adventitia = P_9G_1-B-NC). In the x_θ plots, red and blue signify clockwise and anticlockwise displacement, respectively. In the σ_θ plots, tensile stress concentrations are highlighted by black dotted circles and compressive stress concentrations are highlighted by white dotted circles. Data for the tied model are displayed in Figure S6.

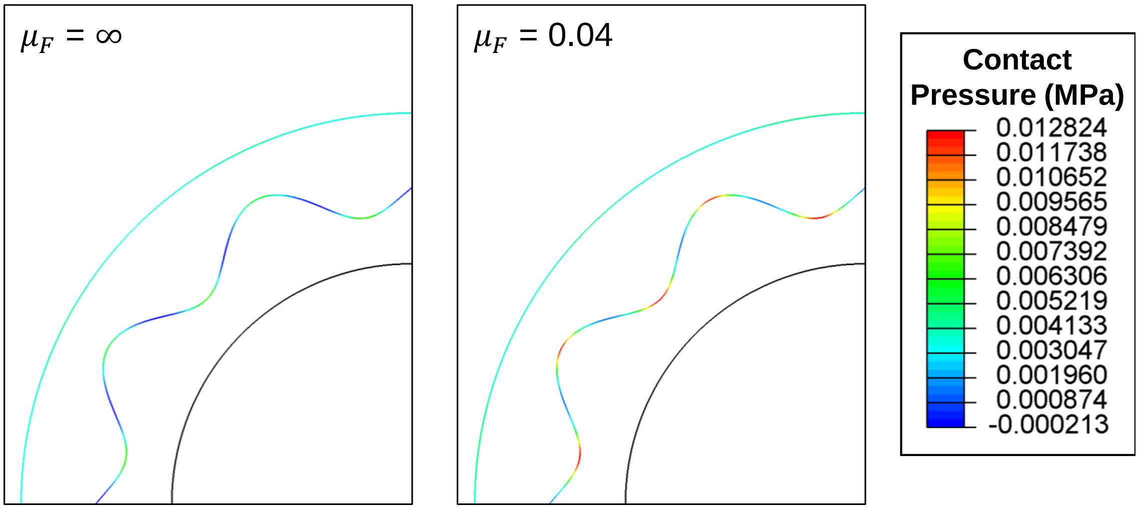

Figure S7: Contact pressures observed at the interface at systolic pressure for rough friction and friction coefficient models.

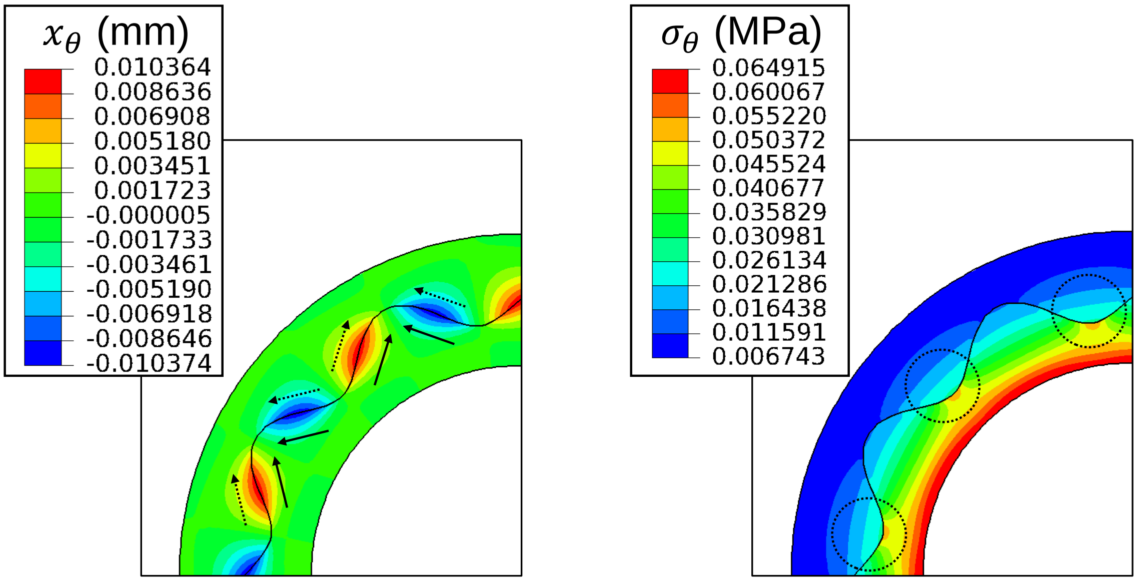

Figure S6: x_θ (left) and σ_θ (right) in the tied surface model (IM = P_9G_1-A-C, adventitia = P_9G_1-B-NC) at systolic pressure. In the x_θ plots, red and blue signify clockwise and anticlockwise displacement, respectively. In the σ_θ plots, tensile stress concentrations are highlighted by black dotted circles and compressive stress concentrations are highlighted by white dotted circles.

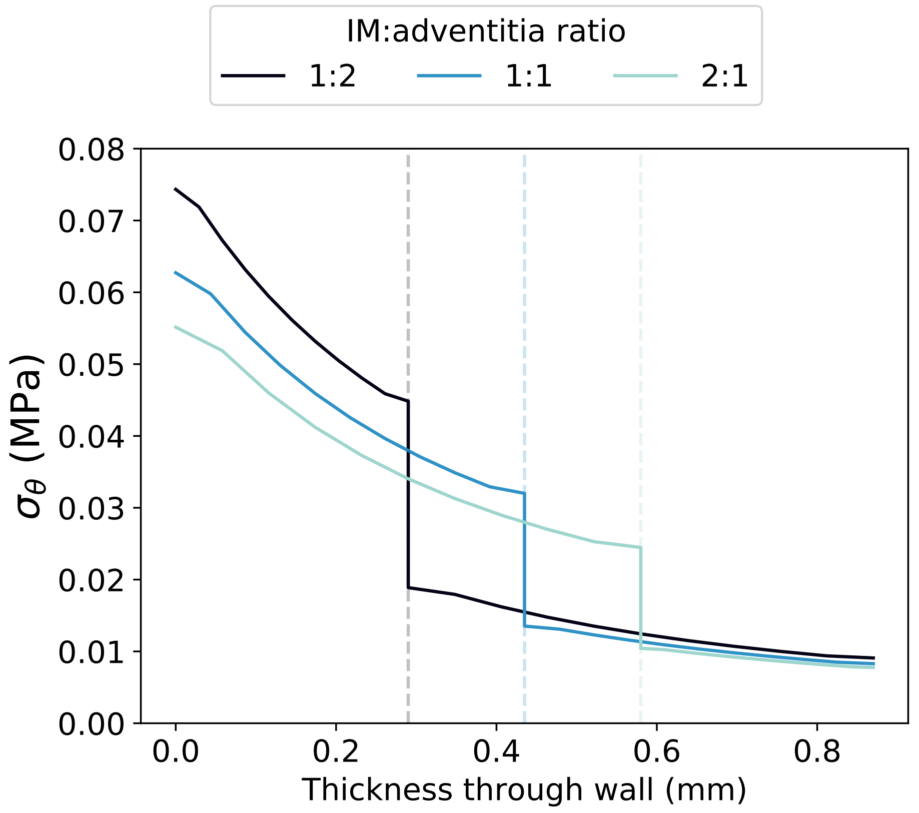

Figure S8: σ_θ distribution at systolic pressure at an IM:adventitia ratio of 1:2, 1:1 and 2:1. Dashed lines show the interface boundary (r).

S3 Varying the Interface Radius of Laminated Grafts: Stress Distribution

For all laminated grafts, (as shown in Figure 2 in the main article) the maximum σ_θ was observed at the luminal surface (Figure S8). A stepwise decrease in σ_θ was observed at the IM-adventitia interface, reflecting the discontinuous nature of the boundary. Increasing the relative volume of the stiffer IM layer increased the distance between the interface and the applied pressure. This influenced the σ_θ distribution in two ways. Firstly, the maximum σ_θ observed at the lumen decreased: $\sigma_\theta = 0.074$ MPa and 0.055 MPa at IM:adventitia ratios of 1:2 and 2:1, respectively. Secondly, the magnitude of σ_θ reduction at the IM-adventitia interface also decreased: at the same IM:adventitia ratios, σ_θ decreased across the interface by 0.026 MPa and 0.014 MPa.

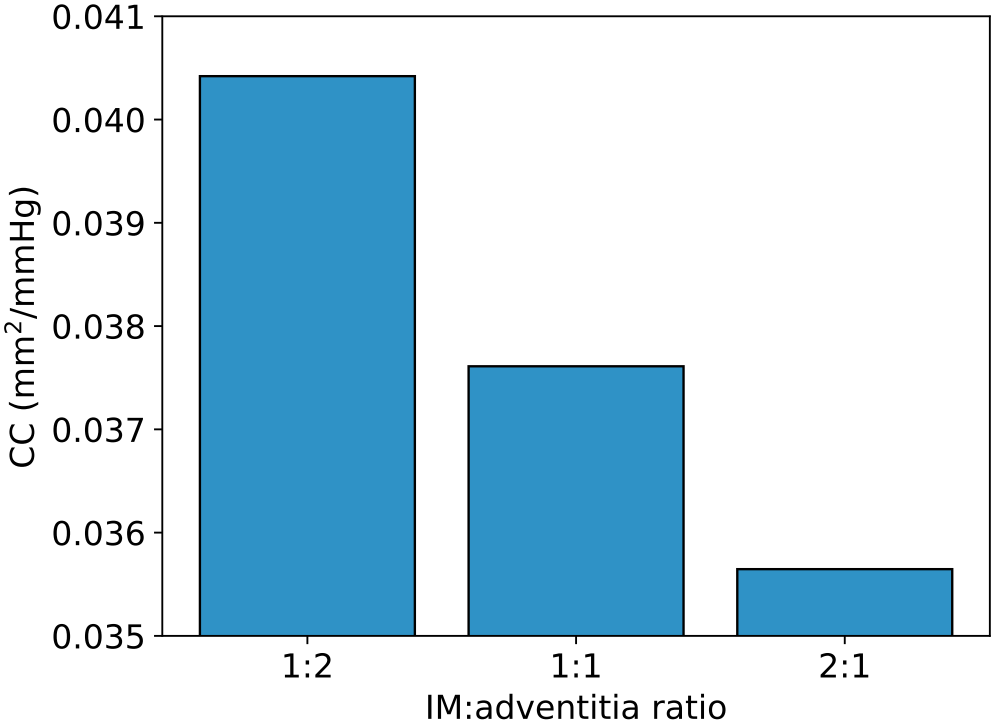

Figure S9: The impact of varying the IM:adventitia ratio on the CC of the graft.

S4 Varying the Interface Radius of Laminated Grafts: Compliance

Increasing the IM:adventitia ratio decreased the CC of the graft (Figure S9). Overall graft stiffness increased with increasing volume of the stiffer IM layer; when IM:adventitia = 1:2, $CC = 0.0356$ mm^2/mmHg. Similarly, the reverse effect was achieved by reducing the volume of the IM; when IM:adventitia = 2:1, $CC = 0.0404$ mm^2/mmHg.

S5 The Circumferential Composition of the IM and Adventitia

Table S1: The areas of the IM and adventitia through circumferential segments 1-10 as a function of A.

A = 0.05 mm		
**Segment**	**Total area of the IM (mm^2)**	**Total area of the adventitia (mm^2)**
**1**	0.078	0
**2**	0.083	0
**3**	0.087	0
**4**	0.092	0
**5**	0.079	0.018
**6**	0.018	0.083
**7**	0	0.106
**8**	0	0.111
**9**	0	0.116
**10**	0	0.121
**1-10 (total)**	**0.437**	**0.555**

A = 0.10 mm		
**Segment**	**Total area of the IM (mm^2)**	**Total area of the adventitia (mm^2)**
**1**	0.078	0
**2**	0.083	0
**3**	0.087	0
**4**	0.091	0.002
**5**	0.063	0.034
**6**	0.035	0.066
**7**	0.002	0.105
**8**	0	0.111
**9**	0	0.116
**10**	0	0.121
**1-10 (total)**	**0.439**	**0.555**

A = 0.15 mm		
**Segment**	**Total area of the IM (mm^2)**	**Total area of the adventitia (mm^2)**
**1**	0.078	0
**2**	0.083	0
**3**	0.087	0
**4**	0.079	0.013
**5**	0.058	0.039
**6**	0.041	0.061
**7**	0.015	0.091
**8**	0	0.111
**9**	0	0.116
**10**	0	0.121
**1-10 (total)**	**0.441**	**0.552**

A = 0.20 mm		
**Segment**	**Total area of the IM (mm^2)**	**Total area of the adventitia (mm^2)**
**1**	0.078	0
**2**	0.083	0
**3**	0.084	0.003
**4**	0.068	0.025
**5**	0.055	0.042
**6**	0.044	0.058
**7**	0.029	0.077
**8**	0.004	0.108
**9**	0	0.116
**10**	0	0.121
**1-10 (total)**	**0.445**	**0.550**

A = 0.25 mm		
**Segment**	**Total area the IM (mm^2)**	**Total area of the adventitia (mm^2)**
**1**	0.078	0
**2**	0.083	0
**3**	0.074	0.013
**4**	0.062	0.030
**5**	0.054	0.043
**6**	0.045	0.057
**7**	0.034	0.072
**8**	0.016	0.095
**9**	0	0.116
**10**	0	0.121
**1-10 (total)**	**0.446**	**0.547**

Table S2: The ratio of IM:adventitia through each circumferential segment as a function of A. The relative areas of the IM and adventitia are normalised to the total area of the corresponding segment. Segment 1 is closest to the lumen, while segment 10 is furthest away from the lumen.

	**IM:adventitia ratio**				
**Circumferential segment**	$\boldsymbol{A}$ **= 0.05 mm**	$\boldsymbol{A}$ **= 0.10 mm**	$\boldsymbol{A}$ **= 0.15 mm**	$\boldsymbol{A}$ **= 0.20 mm**	$\boldsymbol{A}$ **= 0.25 mm**
**1**	1:0	1:0	1:0	1:0	1:0
**2**	1:0	1:0	1:0	1:0	1:0
**3**	1:0	1:0	1:0	0.966:0.034	0.851:0.149
**4**	1:0	0.978:0.022	0.859:0.141	0.731:0.269	0.674:0.326
**5**	0.814:0.186	0.649:0.351	0.598:0.402	0.567:0.433	0.557:0.443
**6**	0.178:0.822	0.347:0.653	0.402:0.598	0.431:0.569	0.441:0.559
**7**	0:1	0.019:0.981	0.142:0.858	0.274:0.726	0.321:0.679
**8**	0:1	0:1	0:1	0.036:0.964	0.144:0.856
**9**	0:1	0:1	0:1	0:1	0:1
**10**	0:1	0:1	0:1	0:1	0:1

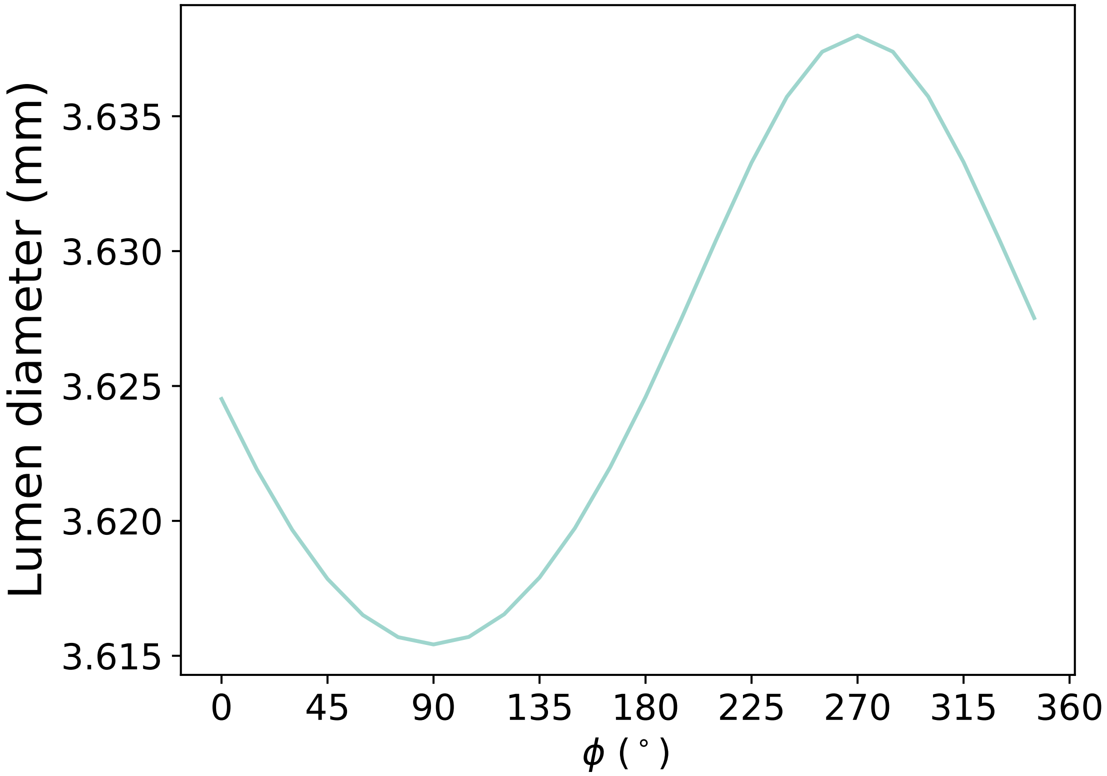

Figure S10: Lumen diameter varies as a function of ϕ. In this example, r = 1.815 mm, A = 0.25 mm and ω = 10.

Table S3: Peak-to-trough height profiles at the lumen surface as a function of A at systolic pressure.

	A = 0.05 mm	A = 0.10 mm	A = 0.15 mm	A = 0.20 mm	A = 0.25 mm
**Peak-to-trough height profile (μm)**	2.12	4.15	6.24	8.56	11.29

S6 Profile of the Lumen

Consequently, the presence of a sinusoidal interface - and therefore non-uniform radial deformation - induced a surface wave on the lumen of the graft. In lieu of surface roughness calculations, r_{lumen} was used to indirectly measure the height profile at the lumen surface. The peak-to-trough height profile increased with increasing A, ranging from 2.12 μm when A = 0.05 mm at systolic pressure to 11.29 μm when A = 0.25 mm at the same pressure (Table S3).

Table S4: Peak-to-trough height profiles at the lumen surface as a function of ω at systolic pressure.

	$\omega = 6$	$\omega = 10$	$\omega = 20$	$\omega = 30$
**Peak-to-trough height profile (μm)**	8.41	4.15	0.68	0.20
